# Dominance of Mating Type A1 and Indication of Epigenetic Effects During Early Stages of Mating in *Phytophthora infestans*

**DOI:** 10.3389/fmicb.2020.00252

**Published:** 2020-02-21

**Authors:** Georgios Tzelepis, Kristian Persson Hodén, Johan Fogelqvist, Anna K. M. Åsman, Ramesh R. Vetukuri, Christina Dixelius

**Affiliations:** Department of Plant Biology, Uppsala Biocenter, Linnean Center for Plant Biology, Swedish University of Agricultural Sciences, Uppsala, Sweden

**Keywords:** *Avrblb2*, epigenetics, heterothallism, mating, ploidy

## Abstract

The potato late blight pathogen *Phytophthora infestans* has both an asexual and a sexual mode of reproduction. In Scandinavia, the pathogen is reproducing sexually on a regular basis, whereas clonal lineages dominate in other geographical regions. This study aimed at elucidating events or key genes underlying this difference in sexual behavior. First, the transcriptomes of eight strains, known as either clonal or sexual, were compared during early stages of mating. Principal component analysis (PCA) divided the samples in two clusters A and B and a clear grouping of the mating samples together with the A1 mating type parents was observed. Induction of genes encoding DNA adenine N6-methylation (6mA) methyl-transferases clearly showed a bias toward the cluster A. In contrast, the *Avrblb2* effector gene family was highly induced in most of the mating samples and was associated with cluster B in the PCA, similarly to genes coding for acetyl-transferases, which play an important role in RXLR modification prior to secretion. *Avrblb2* knock-down strains displayed a reduction in virulence and oospore formation, suggesting a role during the mating process. In conclusion, a number of gene candidates important for the reproductive processes were revealed. The results suggest a possible epigenetic influence and involvement of specific RXLR effectors in mating-related processes.

## Introduction

*Phytophthora infestans* is one of the most devastating plant pathogens, infecting many species in the *Solanaceae* family, particularly potato and tomato. It is a fungal-like, filamentous pathogen, which belongs to Oomycota. Its leading role as one of the most notorious plant pathogens worldwide is associated with its rapid adaptation to fungicides and newly introduced plant resistance genes (Fry, 2008). This high speed of adaptation can be explained by an efficient and mixed reproduction system, and the large amount of effector genes and transposable elements in the *P. infestans* genome (Fry, 2008; Haas et al., 2009). *Phytophthora infestans* is characterized by asexual, parasexual and sexual reproduction systems that together generate a high evolutionary potential (Smoot et al., 1958; Galindo and Gallegly, 1960; Judelson and Yang, 1998). Mating in *P. infestans* requires the presence of A1 and A2 mating types, leading to zygote formation within the developing oospore (Goodwin and Drenth, 1997). Oospores can serve as survival structures, due to their thick cell walls, being dormant in soil for several years and also under harsh environmental conditions (Fay and Fry, 1997; Turkensteen et al., 2000), and they are an efficient source to the new cycle of infection for the coming growing seasons (Fernandez-Pavia et al., 2004; Grünwald and Flier, 2005). In Sweden and Finland, oospores can develop on surviving tubers in the soil and on volunteer plants in the next season (Andersson et al., 1998; Lehtinen and Hannukkala, 2004). In the late 20th century, the A1 mating type was dominating and A2 strains were present only in Mexico (Gallegly and Galindo, 1958). In Europe, the first report of A2 strains came in 1984 (Hohl and Iselin, 1984). In the United States, the dominating US-1 lineage (A1) was replaced by the US-8 lineage (A2) at about the same time-point (Fry et al., 1993). Migration and shipment of potato tubers have now resulted in a spread of the two mating types worldwide, making the *P. infestans* sexual reproduction possible in many regions (Grünwald and Flier, 2005). However, despite the presence of the two mating types, replacement of different virulent lineages is commonly leading to dominance for one genotype (Cooke et al., 2012). Clonal linages have therefore dominated the *P. infestans* populations in many geographical regions and over long time periods (Goodwin and Fry, 1994; Goodwin, 1997; Fry, 2008).

Still many molecular aspects of the sexual reproduction and emergence of clonal lineages of *P. infestans* remain unclear. In Europe, the A1 mating type has been dominating in the United Kingdom, France and Switzerland for many years. A shift to A2 dominance has recently been observed. For example, the displacement of other strains by the highly aggressive Blue-13 (also named 3928A) strain, which has acquired tolerance to phenylamide fungicides, has rapidly occurred during the past years (Cooke et al., 2012). A1 populations are present alongside Blue-13 under field conditions but no hybridization between them has been reported. In the Nordic countries, the situation is strikingly different. High genetic variability is present in the *P. infestans* populations, explained by a high frequency of mating, but no dominating clonal linage has been reported so far (Brurberg et al., 2011; Sjöholm et al., 2013). The mating process of *P. infestans* is more complicated compared to heterothallic fungal species and many aspects of it are still unknown. It has been demonstrated that the inheritance of the mating-type (*MAT*) locus does not follow Mendelian segregation patterns (Fabritius and Judelson, 1997). Rearrangements are common in this chromosomal region and the *MAT* locus is suggested to be hemizygous (Judelson, 1996; Randall et al., 2003; van der Lee et al., 2004).

To expand our knowledge on events explaining the outcome of different activities at the early stages of mating, total RNA from contrasting *P. infestans* strains collected in Sweden, the Netherlands, and the United Kingdom were prepared from *in vitro* cultures and sequenced. A clear bias in gene expression toward the A1 parental strains was observed among the samples. Several interesting genes, such as those coding for adenine methyl-transferases and histone modifiers, followed the same expression patterns. Members of the *Avrblb2* effector gene family were highly induced, particularly in the Dutch mating sample. *Avrblb2* knock-down lines showed reduction in oospore production, indicating a role in sexual reproduction. The impact of epigenetic factors influencing mating process is further discussed.

## Materials and Methods

### *Phytophthora infestans* Strains, Mating and Culture Conditions

**FIGURE 1:**
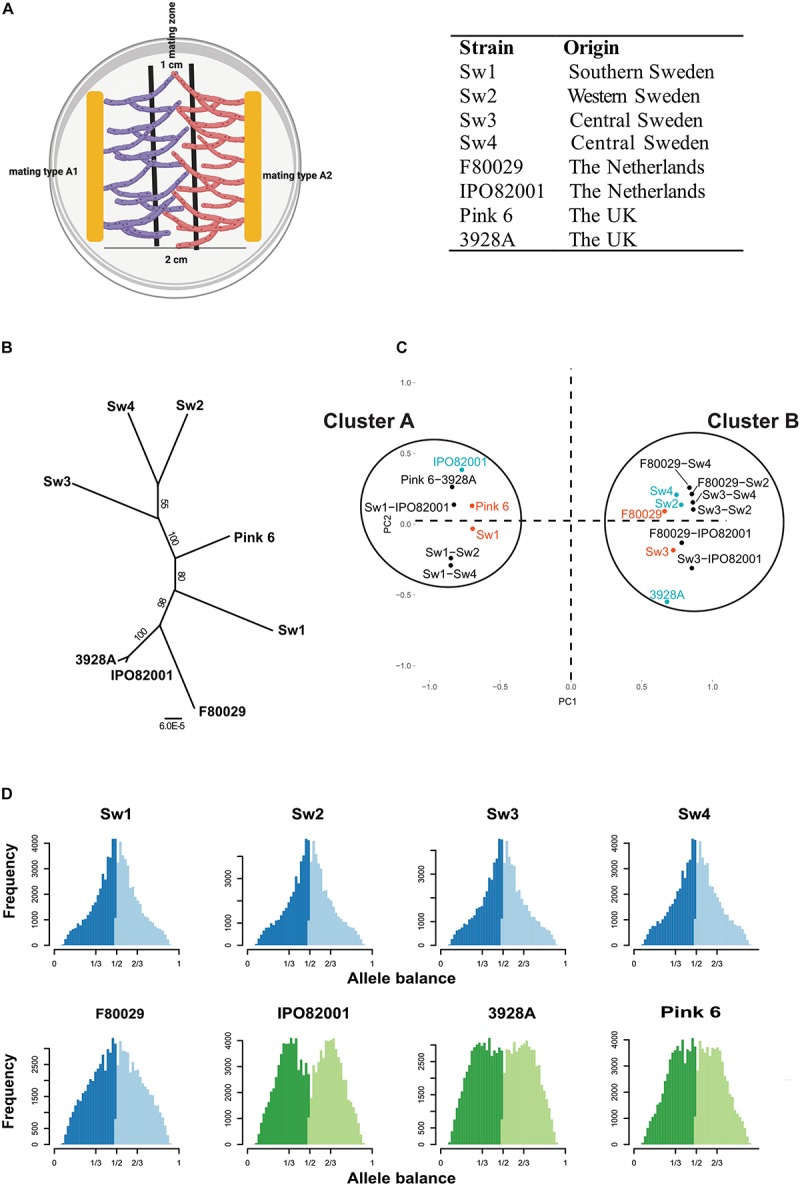
Characterization of *P. infestans* strains with the following mating types; A1: Sw1, Sw3, F80029, Pink 6. A2: Sw2, Sw4, IPO82001, 3928A. **(A)** Illustration on how material for RNA-seq analysis was collected. Mycelia for the mating zone (1 cm) between strains with opposite mating types (A1 or A2) were harvested 7 days post inoculation. **(B)** Phylogeny of *P. infestans* isolates based on SNP data, implemented with the RAxML v8.2X software, using the GTR + Γ model and automatic rapid bootstrapping. Numbers at the nodes indicate bootstrap values. Bar indicates number (6 × 10^– 5^) of substitutions per site. **(C)** Principal component analysis based on significantly differentially expressed genes during mating and in parental strains, grown individually, and normalized against *P. infestans* strain 88069. Red = mating type A1 strains. Blue = mating type A2 strains. Black = mating samples. **(D)** Ploidy analysis of *P. infestans* isolates, based on SNP data. Allele balance of 1/2 = diploid strain. Allele balance of 1/3 and 2/3 = triploid strain. Sw1, Sw2, Sw3, and Sw4 are Swedish isolates, F80029 and IPO82001 are Dutch, Pink6 and 3928A are British. Blue color show diploid strains and green color triploid strains.

All *P. infestans* strains were grown on rye-pea agar (RPA) medium (Caten and Jinks, 1968) supplemented with 2% sucrose and rifampicin (30 μg/ml) and maintained at 20°C in darkness. Eight *P. infestans* strains from potato were included in the mating experiments; four from Sweden (Sw1_A1, Sw2_A2, Sw3_A1, and Sw4_A2), two from the Netherlands (F80029_A1 and IPO82001_A2) and two from the United Kingdom (Pink 6_A1 and Blue 13/3928A_A2). The mating type of the above strains was confirmed by crossing them with tester strains, and self-fertilization was also investigated. In total, 10 mating samples were prepared; four Swedish one Dutch, one British and four “mixed” samples between Swedish and Dutch strains used to investigate whether there is any specific impact of Swedish strains during mating. Mating was promoted on plates where agar strips of A1 and A2 strains were placed in parallel, 2 cm apart, on RPA media, during the same growth conditions as described above (Figure 1A). After 7 days, material was collected from a 1 cm wide mating zone along the meeting point of the different combinations and used for total RNA extraction. The mating zone predominately contained swollen structures of hyphae, along with a mix of few antheridia and oogonia, grown on polycarbonate membrane. Parental strains and the 88069 strain were grown separately and treated exactly as the mating samples. For oospore formation, mating and parental samples were grown as described above for RNA extraction. Materials from the mating zones were harvested 14 days post inoculation, suspended in 20 ml H_2_0 and oospores were counted using hemocytometer.

### Bioinformatic Analyses

Total RNA was extracted from the collected material using the Qiagen Plant mini kit (Qiagen), according to the manufacturer’s instructions. Three biological replicates were taken for each mating sample, and two for each parental sample. In total, 48 RNA strand-specific libraries were generated and sequenced using Illumina HiSeq 2500 at the SNP&SEQ Technology Platform, Science for Life Laboratory at Uppsala University, Sweden. The work flow describing major steps in the analysis is visualized in Supplementary Figure S1. All RNA-seq reads were cleaned for adaptor sequences and low quality reads using Trimmomatic v0.32 (Bolger et al., 2014). The filtered datasets were mapped to the *P. infestans* reference genome (Haas et al., 2009, NCBI accession number PRJNA17665) using Tophat v2.0.10 (Kim et al., 2013). Novel transcripts were discovered using Cufflinks v2.2.1 (Trapnell et al., 2010) and transcript mapping was assessed using HTSeq count v0.6.1 (Anders et al., 2015) with default settings, allowing no mismatches (Supplementary Table S1). Differential expression analysis was performed using DESeq2 v1.8.1 (Love et al., 2014). Two different normalization procedures were applied for the further analysis. Data from strain 88069 was used for the first data normalization, on which the principal component analysis was based. To analyze the mating specific variation, a second normalization was done, where each mating sample was normalized to both its parental strains, grown separately. Significant genes, showing differential expression between at least three of the Swedish matings and the British or Dutch ones, were selected as the main dataset in the Gene Ontology (GO) enrichment analysis (R-package topGO v2.24.0, Alexa and Rahnenführer, 2016). As background dataset, for the GO enrichment analysis, 1,720 genes with similar expression patterns as the main dataset were selected with Genefinder, R-package Genefilter v1.54.2 (Gentleman et al., 2016). GO-terms were inferred from FungiDB v3.0 (Stajich et al., 2012). For single nucleotide polymorphism (SNPs) analysis, duplicated reads were marked using Picard v1.137, GATK v3.3.0 SplitNCigarReads and Indel Realignment (McKenna et al., 2010). Variants were discovered using GATK HaplotypeCaller (de Pristo et al., 2011) and filtered using GATK VariantFiltration. All GATK tools were used with recommended settings for RNA sequencing standard workflow (van der Auwera et al., 2013). Variants were annotated using SnpEff v4.0 (Cingolani et al., 2012). For each protein-coding gene, a nucleotide sequence alignment with the eight samples was made with the discovered SNPs inferred. Genes lacking more than 50% sequence information, for more than four samples, were omitted from further analysis. A phylogenomic analysis was made on the concatenation of the remaining genes using RAxML v8.2X (GTR + Γ, automatic rapid bootstrap) (Stamatakis, 2014). A ploidy analysis was run based on the SNP data and displayed using the vcfR package (Knaus and Grünwald, 2018), filtering away the 10% quantile. The SNP variants, unique to each parent, were used to estimate the parental RNA content of each mating sample. To calculate the ratio of reads in the mating sample in relation to the parental sample, a linear equation was used (*r* = *m*/*p*, where*r* = ratio, *p* = number of reads in the parental sample, *m* = number of reads in the mating sample) implemented in the R package matlib tool (Friendly et al., 2018). The distribution of the ratio for all used SNP variants was thereafter estimated for each mating sample, excluding SNP variants with read depth less than 100 and SNP variants with missing data for the mating sample. To filter for outliers, the 10 and 90% quantiles were removed.

### Validation of RNA-Seq Data

The RNA-seq data were validated using qRT-PCR assay. 1000 ng of total RNA, treated with DNase I (Thermo Fisher Scientific), was reverse transcribed using iScript cDNA synthesis kit (Bio-Rad). qRT-PCR analysis was conducted as described previously (Tzelepis et al., 2012). Primers were designed from predicted exons and listed in Supplementary Table S2, and expression of genes was normalized by actin (*actA*) (Vetukuri et al., 2012). SNP analysis on *actA* gene (*PITG_15117*) (Supplementary Figure S2) showed that primers were located at regions identical to all the studied strains. Relative expression values were calculated from the threshold cycle (Ct) values by using the 2^–ΔΔCT^ method (Livak and Schmittgen, 2001). Transcription patterns of the *Avrblb2* genes were investigated on the same samples as used for RNA-seq analysis. Primers are listed in Supplementary Table S2. Normalization and expression analysis were performed as described above for RNA-seq validation.

### Plasmids Construction, *P. infestans* Transformation and Oospore Production

For hairpin vector construction, the *Avrblb2* family genes were cloned with sense and antisense copies, separated by a 71-nt intron from the *Ste20*-like gene also derived from *P. infestans* (Ah-Fong et al., 2008). Both copies were amplified from the *P. infestans* F80029 genomic DNA, using the primers listed in Supplementary Table S2 and Phusion DNA polymerase (Thermo Fisher Scientific). PCR products were digested (*Fse*I and *Sbf*I for sense; *Sac*II and *Asc*I for antisense) and ligated into the pFTORA vector (Vetukuri et al., 2012), driven by the *ham34* promoter (Judelson et al., 1992). The orientation and integrity of insertions were confirmed by DNA sequencing (Macrogen Inc.). Transformation of *P. infestans* (F80029 strain) was performed as described by Vetukuri et al. (2011). Transcription levels of the *Avrblb2* genes in *P. infestans* knock-down (KD) transformants were investigated using qRT-PCR analysis, as described above. Three independent KD transformants, showing similar growth rate and morphology, were used for functional studies.

*Phytophthora infestans* wild type F80029 strain (WT) and KD transformants in F80029 background were used for leaf inoculations. Leaves, derived from 3 to 4 weeks old Bintje and Bionika potato plants, were inoculated with 100,000 sporangia, while leaves, inoculated with water, were used as control samples. *P. infestans* DNA was extracted using the DNesay Plant Mini kit (Qiagen), and pathogen biomass was quantified in infected potato plants by qPCR, as described above for the qRT-RCR, using the *P. infestans* actin (*actA*) gene, normalized with the elongation factor gene (*elf-1*) from the plant. Primers are listed in Supplementary Table S2. The oospore production was evaluated in crossings *in vitro* on RPA media between the *Avrblb2* KD transformants and the IPO82001 isolate (A2), compared to the WT (F80029) × IPO82001. Microscope slides, containing a thin layer of RPA, were used, and oospore production was counted at the meeting points, 5 and 10 days after mating. Oospores were calculated in the same area in all samples using a microscope (Zeiss Axioplan) and numbers were converted to oospores per mm^2^. For detached leaf and oospore production assays, at least three biological replicates and three independent KD transformants were used. The experiments were repeated twice. For the statistical analysis, the ANOVA (one-way) was conducted, using a General Linear model implemented in SPSS 20 (IBM), performing the Tukey’s or Student’s *T* tests at the 95% significance level.

## Results

### The A1 Genotype Displays Dominance Over the A2 During Mating

In this study we used strains from three European countries; four Swedish (Sw1–Sw4) from different regions (Supplementary Figure S3), two Dutch (IPO82001, F80029), and two British (Pink 6, 3928A). These eight strains had contrasting mating types (A1: Sw1, Sw3, F80029, Pink 6 and A2: Sw2, Sw4, IPO82001, and 3928A), enabling comparisons of events associated to the mating processes. The Dutch mating pair showed reduced oospore production as compared to the Swedish Sw1 × Sw2 and Sw3 × Sw2 samples (Supplementary Figure S4). No oospore production was observed in any parental samples. Total RNA was prepared from mycelia in the mating zones of 10 mating combinations: four Swedish (Sw1 × Sw2, Sw1 × Sw4, Sw3 × Sw2, and Sw3 × Sw4), one Dutch (F80029 × IPO82001), one British (pink 6 × 3928A) and four “mixed” (Sw1 × IPO82001, Sw3 × IPO82001, Sw2 × F80029, and Sw4 × F80029) during early stages of mating and prior to oospore formation. Parental strains were grown separately and treated similar to mating ones.

From Illumina RNAseq data, we first retrieved single nucleotide polymorphism (SNP) information. In phylogenomic analysis three Swedish strains (Sw2, Sw3, and Sw4) grouped together, whereas Sw1 was located closer among the British and Dutch isolates (Figure 1B). A principle component analysis (PCA) based on 10,341 significantly differentially expressed genes (DEGs) normalized against the 88069 strain generated two clusters (Figure 1C). Cluster A included the Sw1, the Pink 6, and the IPO82001 parental strains, and cluster B contained the five remaining parental strains (Sw2, Sw3, Sw4, F80029, and 3928A). All mating samples clustered together with their A1 parental strain. This observation suggests that mating type A1-related gene regulation is dominant during mating. Among the 5,183 DEGs between cluster A and cluster B, 1,602 genes displayed more than doubled expression levels (log2 > 1) between the clusters. This strongly divergent gene subset was slightly biased (868 genes), toward cluster A (Supplementary Figure S5A), whereas 734 genes, showed higher expression for cluster B (Supplementary Figure S5B). Among the 1,602 genes, six (*PITG_18355*, *PITG_07745*, *PITG_21504*, *PITG_14044*, *PITG_03978*, and *PITG_17089*) out of 25 were annotated as basal transcription factors in KEGG^1^. To check for possible biases in the data, an estimation of RNA content in the mating samples was made based on the SNP data (Supplementary Figure S6). No significant biases in RNA content were seen toward any of the two mating types. For example, the Sw1 × Sw2, Sw1 × Sw4, Sw3 × IPO82001 samples were estimated to have similar or slightly higher A2 RNA content, still displaying A1 grouping in the PCA plot (Figure 1C).

It is known that ploidy levels in *P. infestans* strains can vary (Goss et al., 2014; Knaus et al., 2016). Here we inferred the ploidy status of the *P. infestans* strains by exploiting the distribution of read counts at biallelic SNPs. All four Swedish strains and the Dutch F80029 strain displayed a diploid pattern, having an allele balance of 1/2 (Figure 1D). The IPO82001, 3928A, and Pink 6 strains were all triploid (Figure 1D). The results confirm earlier findings that isolates from sexual populations commonly are diploids, whereas clonal linages have a triploid genome constitution (Li et al., 2017). However, the triploids have the ability to change to a diploid genome constitution upon stress, which facilitates sexual reproduction and promotes genetic variation.

### Acetylation and Epigenetic Activity During Mating

**FIGURE 2:**
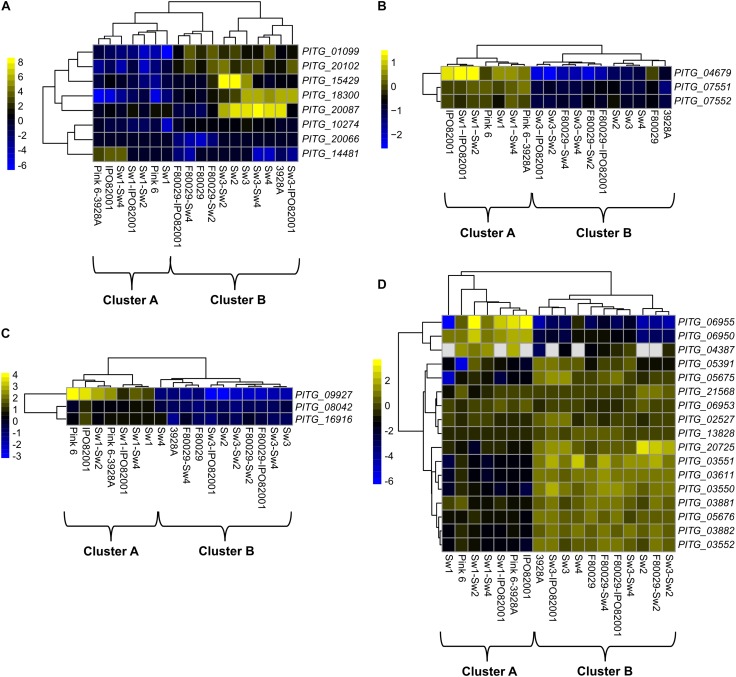
Transcription profiles of selected *P. infestans* genes. **(A)** acetyl-transferase genes, **(B)** adenine N6-methylation (6mA) methyltransferase (*DAMT*) genes, **(C)** histone modification genes and **(D)** genes encoding histone proteins. Data were normalized to the transcriptome of the *P. infestans* 88069 strain (adjusted *p*-value < 0.05, absolute log2 fold change > 2). Yellow and blue colors represent up-regulated or down-regulated genes.

Transcriptome datasets of the ten different *P. infestans* isolate combinations were further dissected. The gene ontology (GO) analysis on all differentially expressed genes showed that only the transferase activity term (GO:0016747) displayed a significant enrichment during mating (Supplementary Table S3). Eight acetyl transferase homologs in *P. infestans* were induced in strains and mating samples (Figure 2A), and followed the B clustering (Figure 1C). Recently, it was shown that DNA methylation in *P. infestans* is regulated by three adenine N6-methylation (6mA) methyltransferase proteins (Chen et al., 2018). The transcription patterns of these genes (*PITG_04679*, *PITG_07551*, and *PITG_07552*) were investigated in our dataset. Interestingly, all three genes showed a bias toward cluster A (Figure 2B). To follow up on this finding, the transcription profile of genes, coding for putative histone modification proteins (*PITG_09927*, *PITG_08042*, and *PITG_16916*), were checked and found to display similar activity patterns, favoring cluster A (Figure 2C). The *PITG_09927* contains multiple Tudor domains, which are recognized methylated histones (Huang et al., 2006; Lee et al., 2008). The *PITG_08042* and *PITG_16916* are putative Tyrosine kinases containing a colony-stimulating factor-1 (CSF-1), which is associated with chromatin (Tagoh et al., 2002). In contrast to the other gene families displayed, three subgroups were observed among the genes coding for histones; genes that were biased toward either cluster A or B, or those being constitutively expressed among the datasets (Figure 2D).

### The Dutch Mating Shows a Different Transcriptome Than the Swedish Ones

**FIGURE 3:**
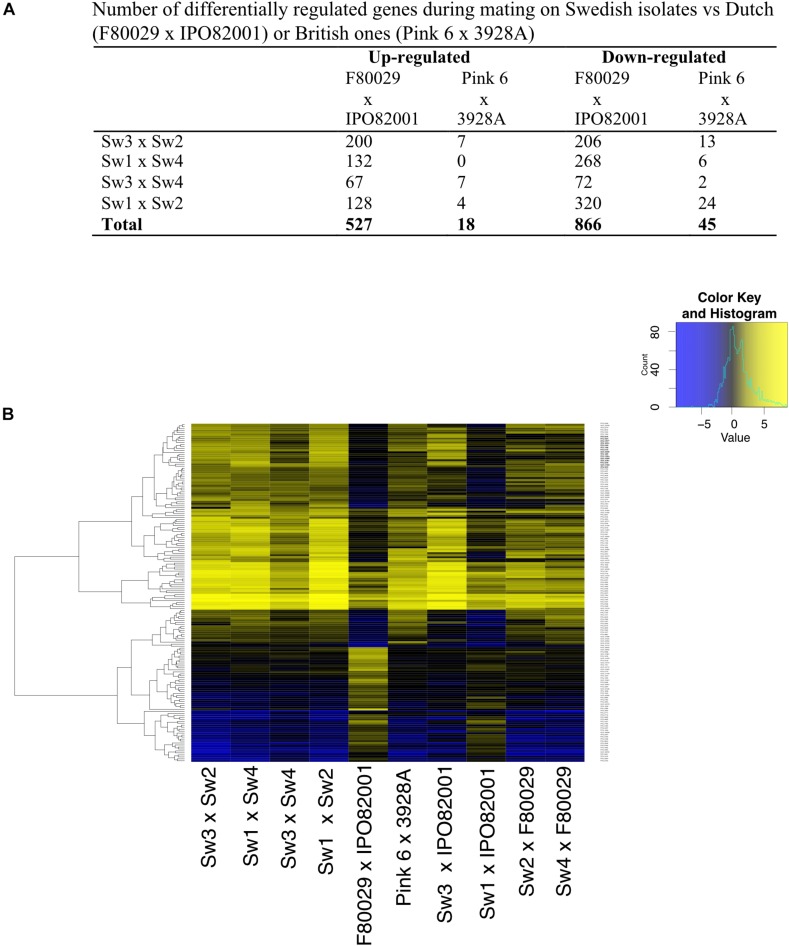
Transcription profiles of *P. infestans* strains during early stages of mating. **(A)** Number of differentially regulated genes in Swedish strains vs. Dutch (F80029 × IPO82001), or British ones (Pink 6 × 3928A). **(B)** Differentially expressed genes in the Dutch, Swedish or British mating samples. Data were normalized to the average expression of both parental strains grown individually (adjusted *p*-value < 0.05, absolute log2 fold change > 2). Yellow and blue colors represent up-regulated or down-regulated genes.

In order to investigate the transcriptome differences in Swedish mating samples versus samples from the Netherlands and the United Kingdom, a second normalization was performed where each mating sample was normalized to both parental strains, grown separately. Data from this analysis revealed that the Dutch (F80029 × IPO82001) and the Sw1 × IPO82001 pairs generated the most divergent patterns compared to the other eight combinations (Figures 3A,B). The expression profiles of selected genes were investigated using qRT-PCR analyses and were in agreement with the RNA-seq data (Figure 3 and Supplementary Figure S7), In total, 527 genes were differentially up-regulated in Swedish vs. Dutch strains, while only 18 in Swedish vs. British mating samples (Figure 3A). Among the genes that showed elevated transcripts in the Dutch pair compared to the Swedish pairs (Sw3 × Sw2, Sw1 × Sw4, Sw3 × Sw4, and Sw1 × Sw2) were genes encoding enzymes that are involved in energy production and metabolism, such as FAD-dependent oxidoreductase (*PITG_03815*) and dehydrogenases (*PITG_00908*, *PITG_10290*) (Supplementary Table S4). Genes that encoded ATP-binding cassette proteins (ABC transporters; *PITG_07717*, *PITG_07716*, *PITG_04787*) were also up-regulated in the Swedish mating samples, whereas *PITG_17956* (the croquemort mating protein M82) was induced in the Dutch mating pair (Supplementary Table S4). Induction of *PITG_17956* has been reported during the early stages of mating in *P. infestans* (Fabritius et al., 2002). Increased transcripts of ABC transporter genes might be related to higher needs of energy and mating hormones during potential mating in the Dutch strains. Elevated levels were observed also for genes that encode hydrolytic enzymes, such as endo-glucanase (*PITG_08944*) and beta-glucosidases (*PITG_01398*, *PITG_01399*, and *PITG_01484*) in the Dutch strains (Supplementary Table S4).

Finally, when comparing the repressed genes, 866 were differentially regulated between Swedish and Dutch mating samples and only 45 in Swedish vs. British ones (Figures 3A,B and Supplementary Table S5). We observed that genes, encoding the mating-induced protein 96 (M96) (*PITG_02519*, *PITG_09067*, *PITG_22707*, *PITG_05905*), had reduced transcript levels in the Dutch mating pair (F80029 × IPO82001). Similar patterns were observed for genes coding for Crinkler effector proteins (*PITG_09043* and *PITG_09053*).

### The Avrblb2 Effector Family Is Induced During Early Stages of Mating

**FIGURE 4:**
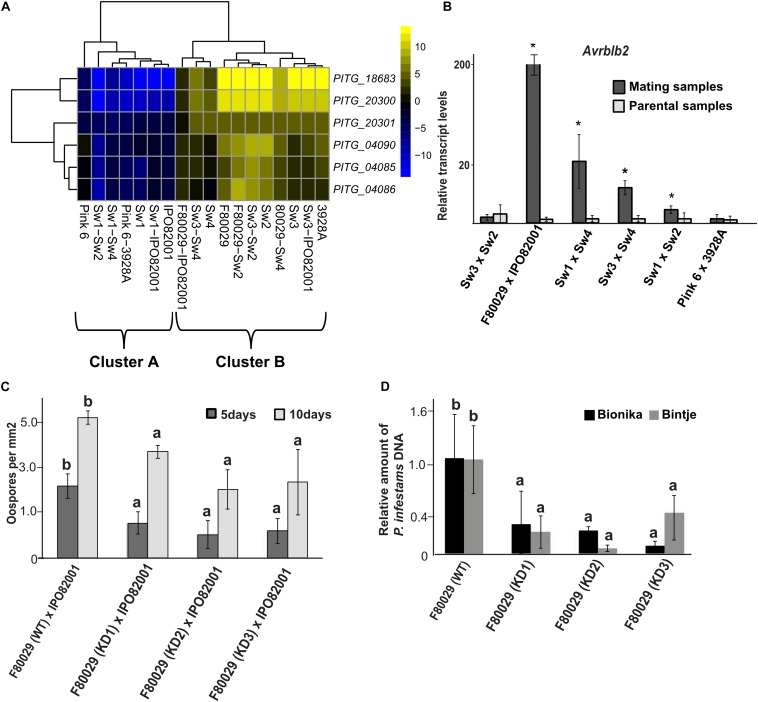
Transcription and phenotypic analysis of *P. infestans. Avrblb2* effector family. **(A)** Heatmap of genes coding for *Avrblb2* effectors. Data were normalized to the transcriptome of the 88069 *P. infestans* isolate (adjusted *p*-value < 0.05, absolute log2 fold change > 2). Yellow and blue colors represent up- or down-regulated genes. **(B)** qRT-PCR analysis of the *Avrblb2* genes during mating compared to parental strains. Data were normalized to the expression of actin (*act*) and calculated, using the 2^–DDCt^ method. Average of both parental strains is shown at the graph. Asterisks (*) indicate statistically significant differences between mating and parental samples according to Student’s *T*-test (*p* < 0.05). **(C)** Quantification of oospore production in F80029 (WT) strain and *Avrblb2* (KD) transformants, crossed with IPO82001 (A2) strain, 5 and 10 days after mating. Different letters (a,b) indicate statistically significant differences between crossings of WT × IPO82001 vs. KD × IPO82001 according to Tukey’s *T* test (*p* < 0.05). **(D)** Quantification of *P. infestans* F80029 strain (WT) and *Avrblb2* (KD) transformants biomass upon infection of Bintje and Bionika leaves, 3 and 4 days post inoculation, respectively. qPCR was conducted in genomic DNA, extracted from infected leaves using the *P. infestans act* gene, and normalized to the potato *elf-1* gene. Error bars represent standard deviation (SD), based on at least three biological replicates. Different letters (a,b) indicate statistically significant differences according to Tukey’s test (*p* < 0.05). For detached leaf and oospore production assays, at least three biological replicates and three independent KD transformants were used. The experiments were repeated twice.

Four genes, coding for RXLR effector proteins, were highly induced in the Dutch mating pair. These were *Avr2* (*PITG_22870*), *Avrblb2* (*PITG_04085*, *PITG_04086*), and *PITG_12057* (Supplementary Table S4). The *Avrblb2* is a family of seven paralogs sharing more than 90% sequence similarity (Supplementary Figure S8), and the *PITG_04085* and *PITG_04086* are antisense copies oriented in a head-to-head configuration (Oliva et al., 2015). SNP analysis on *Avrblb2* genes showed one nucleotide insertion or deletion in some stains (Supplementary Figure S9). Further analysis on *Avrblb2* genes, using the transcriptome of the isolate 88069 as a reference, revealed that induction of these genes, in parental and mating samples (Figure 4A), grouped to cluster B in the PCA plot, similar to acetyltransferase expression patterns (Figure 2A). N-terminal acetyltransferases play an important role in acetylation of effectors before secretion and cleavage of the RXLR motif in *P. infestans*, as exemplified by AVR3a^EM^ (Wawra et al., 2017). Validation by qRT-PCR analysis revealed that *Avrblb2* was highly induced, not only in the Dutch strain mating (F80029 × IPO82001), but also in two of the Swedish samples (Sw3 × Sw2 and Sw3 × Sw4) as compared with the parental samples (Figure 4B). No up-regulation of this effector was observed during mating, between the British strains (Figure 4B).

The *Avrblb2* family genes were knocked down (KD), using a hairpin construct, and the role of this effector was studied in virulence and oospore formation. Three independent transformants in the F80029 background (mating type A1), displaying stable resistance to geneticin, were selected, and all of them showed significantly reduced expression levels of the *Avrblb2* genes (Supplementary Figure S10). Any attempt to knock-down the *Avrblb2* homolog in the IPO82001 strain continuously failed. The potential impact of the Avrblb2 effector in reproduction was investigated, using crosses of the KD transformants in F80029 genomic background with the IPO82001 (mating type A2) strain. Oospore production was measured at two time points, 5 and 10 days after mating. Reduced oospore production was observed at both time points, supporting a potential role of this effector category in sexual reproduction (Figure 4C). The role of the Avrblb2 effectors in *P. infestans* virulence was also evaluated on two potato cultivars, with variable resistance levels. Bintje is a highly susceptible cultivar, while Bionika harbors the *Rpi-blb-2* gene introduced from *Solanum bulbocastanum* (Song et al., 2003; van der Vossen et al., 2005). Pathogen biomass was quantified upon infection with F80029 or the three KD transformants applied individually and we observed that the silenced lines displayed reduced biomass on infected plant tissue compared with the wild type in both cultivars (Figure 4D).

## Discussion

The overall aim of the current work was to advance the understanding of mechanisms explaining differences of mating capacity at the early stages between different European *P. infestans* strains. To address this question we compared British, Dutch and Swedish strains on transcriptome levels to elucidate potential differences between mating-associated gene activities. Based on SNP data, we found that the Sw1 genotype was more similar to the Dutch and British strains than to the three other Swedish strains. The comparative transcriptomic analysis between Swedish and Dutch or British mating samples showed also that the Sw1 × IPO82001 mating sample behaves more similar to the Dutch mating rather than to other Swedish ones. This result was not completely unexpected, since the Sw1 isolate was collected in the southernmost part of Sweden, and long-distance wind spreading of sporangia, from Central Europe, would facilitate establishment of new *P. infestans* genotypes in this region. When comparing the eight parental *P. infestans* strains, the gene expression patterns differentiated into two groups not linked to mating type or ploidy levels; Sw1, IPO82001, Pink 6 form cluster A and F80029, 3928A, Sw2, Sw3, Sw4 cluster B. We found mating samples in the same groups as the parents, strictly linked to the gene expression of the parental A1 strains, in each combination. The grouping into cluster A and B is stringently seen in 1,602 genes (Supplementary Figure S5). Among those genes, basal transcription factors and epigenetic factors were found, possibly regulating the remaining gene set.

The underlining explanation of the division into frequent sexual reproduction of *P. infestans* strains, as found in Northern Europe versus the clonal lineages in Western Europe, appears to be regulated by complex events. Epigenetic influences during sexual reproduction, such as DNA methylation, histone modification and small RNAs (sRNAs), have been reported in several organisms including plants, animals and fungi (Quadrana and Colot, 2016; Kronholm et al., 2016; Donkin and Barrès, 2018). So far, no evidence of 5-methylcytosine DNA-methylation, which is prevalent in many organisms, has been reported in *P. infestans* (van West et al., 2008). Instead, adenine N6-methylation (6mA) methyltransferases (DAMTs) are shown to generate epigenetic marks across its genome (Chen et al., 2018). The three DAMT genes were also found to be up-regulated in our PCA group. A dataset, in contrast to the different developmental stages of *P. infestans* analyzed by Chen et al. (2018).

Whether epigenetic reprogramming, including transgenerational gene silencing, is a frequently occurring mechanism in the evolution and adaptation to new environments by *P. infestans* requires new in-depth analysis. The high proportion of transposable elements (TEs) in *Phytophthora* genomes and the co-localization of effector genes in TE-rich regions (Haas et al., 2009; Whisson et al., 2012) may drive epigenetic events in *P. infestans* as well. Emerging reports on sexual reproduction in animals and plants have uncovered different active RNA silencing pathways during sporogenesis, gametogenesis and after fertilization, influencing reproductive success. Processes involved are changes in DNA methylation, histone reprogramming, TE reactivation, and involvement of different classes of sRNAs (Martinez and Köhler, 2017; Borges et al., 2018; Martinez et al., 2018). Epigenetic reprogramming in plants also can be triggered by abiotic constrains (Wibowo et al., 2016; Lämke and Bäurle, 2017) and if applicable to *P. infestans*, such a function could generate increased opportunities to adapt to various changes in climate conditions. The extent of genetic variation in Scandinavian *P. infestans* populations has been debated (Maurice et al., 2019). Beside different marker systems employed for the analysis by different research groups, variation in the observations may as well reflect epigenetic influences driven by annual climate fluctuations.

Here, we used our dataset to generate some insights into the activity of annotated histone-modifying genes in *P. infestans*. This strategy demonstrated clear sub-groups among the different samples in our study. We hypothesize that *P. infestans* has evolved its own epigenetic program, acting on sexual reproduction, eventually leading to either clonal or genetically heterogeneous field populations. Dissection of gametes, zygotes, spores and surrounding somatic nurse tissues or cells, in combination with high-through-put sequencing technologies and transgenic lines with silenced target genes, are required to experimentally demonstrate the proposed transgenerational events in mating between different strains of *P. infestans*. However, *P. infestans* is a cumbersome organism to explore this type of advanced molecular functions.

Genes coding for the *Avrblb2* effector family were highly induced during mating in most of our analyzed samples. Similarly, induction of RXLR and Crinkler effector genes has also been seen during mating between three other A1/A2 strains (Niu et al., 2018). The function of Avrblb2 effector, in the plant infection process, is to block the plant C14 protease and prevent its secretion into the apoplast (Bozkurt et al., 2011). In a search of proteases in the *P. infestans* genome, we found a cysteine protease homolog to C14 (*PITG_02030*), a gene with no differential gene expression in our mating datasets (Supplementary Figure S11). Secreted proteases are also known to play important roles in the mating processes of *Saccharomyces cerevisiae*, where they influence the mating pheromones in various ways (Barkai et al., 1998; Jin et al., 2011). In the yeast system the aspartyl protease Bar1, functions as a guide to ensure mating with a suitable partner (Ballensiefen and Schmitt, 1997). In the *P. infestans* genome, we identified a homolog to Bar1, *PITG_10524*, which displayed similar transcription pattern as *Avrblb2* family (Supplementary Figure S11). Whether *P. infestans* shares some of the gene functions related to mating known in yeast remain to be demonstrated.

In this study, events during mating, between different strains of *P. infestans* were monitored on transcriptome levels. A clear dominance of mating type A1 and a potential epigenetic impact on the sexual reproduction was observed. Functional analysis of the Avrblb2 effector suggested a possible role in other types of cell-cell interactions rather than only in plant–pathogen interactions, including those affecting the local microbial community (Snelders et al., 2018). The observations reported here on several new potential mating-associated gene candidates and their regulation form the basis of new, research directions on the biology of *P. infestans*.

## Data Availability Statement

The RNA-seq data used in this study has been deposited in the National Center for Biotechnology Information (NCBI) database, and Gene Expression Omnibus under the accession number GSE94137.

## Author Contributions

CD, RV, AÅ, and GT conceived the experiments. GT, AÅ, and RV performed the experiments. JF and KH conducted the bioinformatics analysis and interpreted the data. All authors contribute to writing of the manuscript and text revision.

## Conflict of Interest

The authors declare that the research was conducted in the absence of any commercial or financial relationships that could be construed as a potential conflict of interest.

## Footnotes

1 https://www.genome.jp/kegg-bin/show_pathway?pif03022
