# Supplementary material for: Dominance of Mating Type A1 and Indication of Epigenetic Effects During Early Stages of Mating in Phytophthora infestans
Source: Front Microbiol. 2020 Feb 21;11:252. doi: 10.3389/fmicb.2020.00252 (PMC7046690; doi:10.3389/fmicb.2020.00252)
Supplement: Supplementary file 1 [file Data_Sheet_1.PDF]

Supplementary Table 1. Number of sequence reads of each sample

Sample	No. of reads	Concordant pair alignment rate	Replicates	Variants	Heterozygous
Sw3	48,912,054	83.70%	2	86,467	52.06%
Sw2	48,233,872	82.80%	2	93,461	50.74%
Sw1	44,374,528	83.00%	2	83,127	51.78%
Sw4	46,850,568	84.00%	2	88,282	50.43%
F80029	51,524,356	86.15%	2	71,420	66.17%
IPO82001	46,028,278	83.95%	2	103,118	66.11%
3928A	49,053,462	84.10%	2	94,036	64.05%
Pink 6	47,366,632	83.60%	2	104,630	62.41%
88069	52,199,592	85.05%	2	92,449	71.60%
Sw3 x IPO82001	70,209,060	83.13%	3		
Sw2 xF80029	90,645,704	83.83%	3		
Sw3 x Sw2	121,322,714	83.77%	3		
F80029 x IPO82001	97,153,540	84.70%	3		
Sw1 x Sw4	92,707,516	83.90%	3		
Sw1 x IPO82001	64,336,452	84.10%	3		
Sw4 xF80029	71,169,628	84.50%	3		
Sw3 x Sw4	146,824,368	83.63%	3		
Sw1 x Sw2	138,515,000	82.67%	3		
3928A x Pink 6	109,900,156	83.03%	3		

Supplementary Table 2. Primer sequences used in the study

Primer name	Forward primer 5'-3'	Reverse primer 5'-3'	Purpose in this study
Pi_Avrblb2	cgc cgt cgc agc att cc	ccc ggc cgc tct gaa taa ctt	qRT-PCR assay
PITG_22870	tgc cgc ccc aag ccc taaa	tta gcc ttg tcc ttc gcc ttt accg	qRT-PCR assay
PITG_08944	atc cct gct act ctt tcc ctc gtc	tgt gca cca gat ttg tcg ttg tc	qRT-PCR assay
PITG_15033	tcc tgc cgg cta caa cga ga	tgg cat ctc aat cag gac gga atc	qRT-PCR assay
PITG_17956	cgg cct cct ttg ctt cca cac taa	ata gcc agc gga tcc agc gag taa	qRT-PCR assay
PITG_01399	gca cca ggg ta caca ggc tca ct	ata ctg atc ctc gcc ttg tct cca	qRT-PCR assay
PITG_15117 (actA)	catc aag gag aag ctgac	gac gac tcg gcg gcag	qRT-PCR assay/ DNA quantification
Elf-1	ttt ggc cct act ggt ttgac	ggg tca tcc ttg gac ttt ga	DNA quantification
***Avrblb2*sense**	atat ggccggcc ctg ttc ttg cct ttg cgg ttt ta	atat cctgcagg ttg gcc gcc tcg ata agt tt	Hairpin construction
***Avrblb2*antisense**	atat ccgcgg ctg ttc ttg cct ttg cgg ttt ta	atat ggcgc ttg gcc gcc tcg ata agt tt	Hairpin construction

Supplementary Table 3. GO analysis of differentially expressed genes on mating samples

GO.ID	Term	P value
**GO:0016747**	**transferase activity. transferring acyl ...**	**0.017**
GO:0008270	zinc ion binding	0.052
GO:0004553	hydrolase activity. hydrolyzing O-glycos...	0.058
GO:0000166	nucleotide binding	0.110
GO:0030246	carbohydrate binding	0.134
GO:0003824	catalytic activity	0.137
GO:0035639	purine ribonucleoside triphosphate bindi...	0.148
GO:0032550	purine ribonucleoside binding	0.159
GO:0008484	sulfuric ester hydrolase activity	0.175
GO:0003774	motor activity	0.175
GO:0043169	cation binding	0.265
GO:0050662	coenzyme binding	0.293
GO:0035091	phosphatidylinositol binding	0.304
GO:0016817	hydrolase activity. acting on acid anhyd...	0.366
GO:0042626	ATPase activity. coupled to transmembran...	0.389
GO:0003723	RNA binding	0.444
GO:0004674	protein serine/threonine kinase activity	0.470
GO:0032559	adenyl ribonucleotide binding	0.473
GO:0032555	purine ribonucleotide binding	0.474
GO:0005215	transporter activity	0.492
GO:0009405	pathogenesis	0.14
GO:0006952	defense response	0.14
GO:0006066	alcohol metabolic process	0.19
GO:0015672	monovalent inorganic cation transport	0.19
GO:0005975	carbohydrate metabolic process	0.23
GO:0044283	small molecule biosynthetic process	0.26
GO:0030001	metal ion transport	0.30
GO:0018130	heterocycle biosynthetic process	0.38
GO:1901362	organic cyclic compound biosynthetic pro...	0.43
GO:0055114	oxidation-reduction process	0.50
GO:0008152	metabolic process	0.52
GO:0034220	ion transmembrane transport	0.52
GO:0006820	anion transport	0.52
GO:0007154	cell communication	0.53
GO:0090407	organophosphate biosynthetic process	0.59
GO:0009117	nucleotide metabolic process	0.59
GO:0043412	macromolecule modification	0.61
GO:0016070	RNA metabolic process	0.61

GO:1901137	carbohydrate derivative biosynthetic pro...	0.65
GO:0050896	response to stimulus	0.69
GO:0005856	cytoskeleton	0.05
GO:0005576	extracellular region	0.19
GO:0016020	membrane	0.31
GO:0016021	integral component of membrane	0.32
GO:0031224	intrinsic component of membrane	0.49
GO:0043234	protein complex	0.54
GO:0032991	macromolecular complex	0.75
GO:0005634	nucleus	0.89
GO:0005737	cytoplasm	0.97
GO:0005622	intracellular	1.00
GO:0005623	cell	1.00
GO:0044464	cell part	1.00
GO:0044422	organelle part	1.00
GO:0043231	intracellular membrane-bounded organelle	1.00
GO:0044424	intracellular part	1.00
GO:0043232	intracellular non-membrane-bounded organ...	1.00
GO:0044425	membrane part	1.00
GO:0044446	intracellular organelle part	1.00
GO:0005575	cellular_component	1.00
GO:0043226	organelle	1.00

Supplementary Table 4. Differential expression in Swedish mating samples as compared to F80029 x IPO82001 (Dutch) or Pink 6 x 3928A (British). Up-regulated genes set.

Gene Access number	Sw3 x Sw2	Sw1 x Sw4	Sw3 x Sw4	Sw1 x Sw2	F80029 x IPO-0	Pink 6 x 3928A
PITG_22870	-1.61273540	-1.52124695	-1.480924393	-0.741563481	1.748848851	-0.329471577
PITG_12057	-1.44137216	-1.142365794	-0.978950762	-1.255516949	0.798810017	-1.05363942
PITG_04086	-1.41792880	-0.822122409	-0.419107	-1.230381896	2.433759419	-1.76377147
PITG_04085	-1.79224566	-0.922243474	-0.815689205	-0.72468616	1.87863556	-1.546660755
PITG_07717	-2.06600894	-1.879312969	-1.256366468	-2.028221936	1.320783573	-1.381480212
PITG_07716	-2.29996570	-2.06400635	-1.205699927	-2.393233718	0.85306158	-1.256957821
PITG_04787	-1.95657611	-1.61259595	-1.36234672	-1.61135762	0.703848691	-0.873662936
PITG_05902	-3.80557963	-1.896500784	-1.593818603	-2.711076143	0.761054504	-2.082043927
PITG_08944	-3.53310527	-1.526412114	-2.173864925	-1.632169291	1.723688242	-1.343177917
PITG_10290	-2.04609333	-1.84929739	-0.744964133	-1.300318216	0.573487661	-0.995214676
PITG_15078	-0.04331864	0.422226005	-0.151860375	0.303182103	2.117266452	0.337269008
PITG_15033	-0.94633464	-1.098335786	-0.296768964	-0.906786972	1.658615298	-0.219088378
PITG_03815	-0.85793138	-1.170348477	-0.425320179	-1.18314984	0.752263005	-1.077951627
PITG_04129	-0.90009097	0.298314733	-0.491708644	0.238440605	3.624251692	0.283099982
PITG_17956	-1.61260420	-1.120233204	-1.252189294	-1.25026558	0.579677119	-1.228275167
PITG_18534	-2.01163714	-2.590364612	-1.294774061	-1.754649915	1.291958994	-0.169490607
PITG_00908	-0.71312186	-0.321496706	-0.321834375	-0.520751753	1.28998036	-0.199599743
PITG_10573	-1.32677228	-0.426713854	-0.204618423	-0.225016326	1.77289757	-0.176542182
PITG_23077	-1.95240474	-1.238635277	-0.629942967	-1.096507026	1.044370758	-1.943385948
PITG_01398	-2.29843076	-1.24300589	-0.684220869	-1.588333254	0.567328165	-1.689396791
PITG_01399	-2.22157867	-1.409166155	-0.772991388	-1.501634008	1.082527661	-1.770012391
PITG_01484	-1.23886679	-0.858854918	-0.441981489	-0.996717558	0.912451398	-0.864361374

Genes have an adjusted p-value < 0.05 and absolute log2 fold-change >2. Data has been normalized to the transcriptome of parental isolates grown individually

Supplementary Table 5. Differential expression of Swedish matings as compared to F80029 x IPO8200 (Dutch) or Pink 6 x 3928A. Down-regulated gene set.

Gene Access number	Sw3 x Sw2	Sw1 x Sw4	Sw3 x Sw4	Sw1 x Sw2	F80029 x IPO-0	Pink 6 x 3928A
PITG_00045	1.031513964	1.336766911	0.69803433	0.97991825	-0.66235612	1.288665567
PITG_00251	2.29052702	4.176037078	0.812456225	5.98805931	-0.49396853	2.769599922
PITG_22670	0.692972226	2.635943971	0.514914118	2.071217	-1.3117164	1.529699646
PITG_09887	2.783762631	2.169731302	1.101183467	4.45965693	-0.51359562	4.451584727
PITG_11140	5.058925459	2.199447684	1.126262174	5.63086414	-0.56516159	3.131401269
PITG_10765	1.447608613	1.047128465	0.898284283	0.93437509	-0.92239039	1.266628193
PITG_10750	2.078547599	1.390833095	1.519174771	0.96752903	-0.71942209	1.14584285
PITG_02505	7.780301935	7.561992489	5.822364125	8.18070387	4.710249548	6.124276951
PITG_12312	3.166284534	2.978553497	1.060777709	3.63063475	-0.28708987	1.951871851
PITG_02504	7.282955324	7.373825728	4.879196792	7.88323542	2.096315816	5.853301723
PITG_02656	6.363562929	5.171055028	3.349959737	7.13959036	1.392478303	3.95899721
PITG_02796	1.717999916	1.172107254	1.518578729	0.88283454	-0.73595549	1.31159464
PITG_14420	1.830306065	2.906789419	1.290336162	3.16231357	-1.62283759	1.162735751
PITG_03815	-0.85793138	-1.170348477	-0.425320179	-1.18314984	0.752263005	-1.077951627
PITG_05579	1.156424576	2.130588431	1.507721743	1.45440510	-0.58954692	0.795299765
PITG_05412	1.23158531	1.575078861	0.354889623	1.20270271	-0.54984105	1.654655875
PITG_06370	7.192670244	6.823965493	4.066710994	7.65952908	0.846920785	5.029291223
PITG_06143	2.812546776	3.302037715	2.09147505	3.42053884	0.617724784	2.524939342
PITG_02099	1.554269997	1.11579972	0.780616548	0.84153118	-0.73557606	0.192236635
PITG_16525	-0.57760145	0.652057936	0.394268619	0.33740340	-1.87338279	2.385864055
PITG_04049	1.278110941	2.871335796	1.044412668	1.76667372	-0.70808158	1.267761057
PITG_09043	2.066293294	3.07473842	0.930458324	3.56852130	-0.18848365	0.957308377
PITG_09053	2.61799331	3.770073122	1.540471295	3.60289819	0.305301437	0.915229312
PITG_02519	7.066740188	6.455651882	3.733552893	7.49889223	2.849088162	3.410024893
PITG_09067	1.658936385	1.851246996	0.892126629	1.90420394	-0.24769395	1.044661476
PITG_22707	4.422561223	3.633418717	1.69379627	4.96298929	1.108588627	1.982461992
PITG_05905	4.45212198	5.840511068	4.148458034	4.90433456	0.448060177	2.123679648

Genes have an adjusted p-value < 0.05 and absolute log2 fold-change >2. Data have been normalized to transcriptome of parental isolates grown individually.

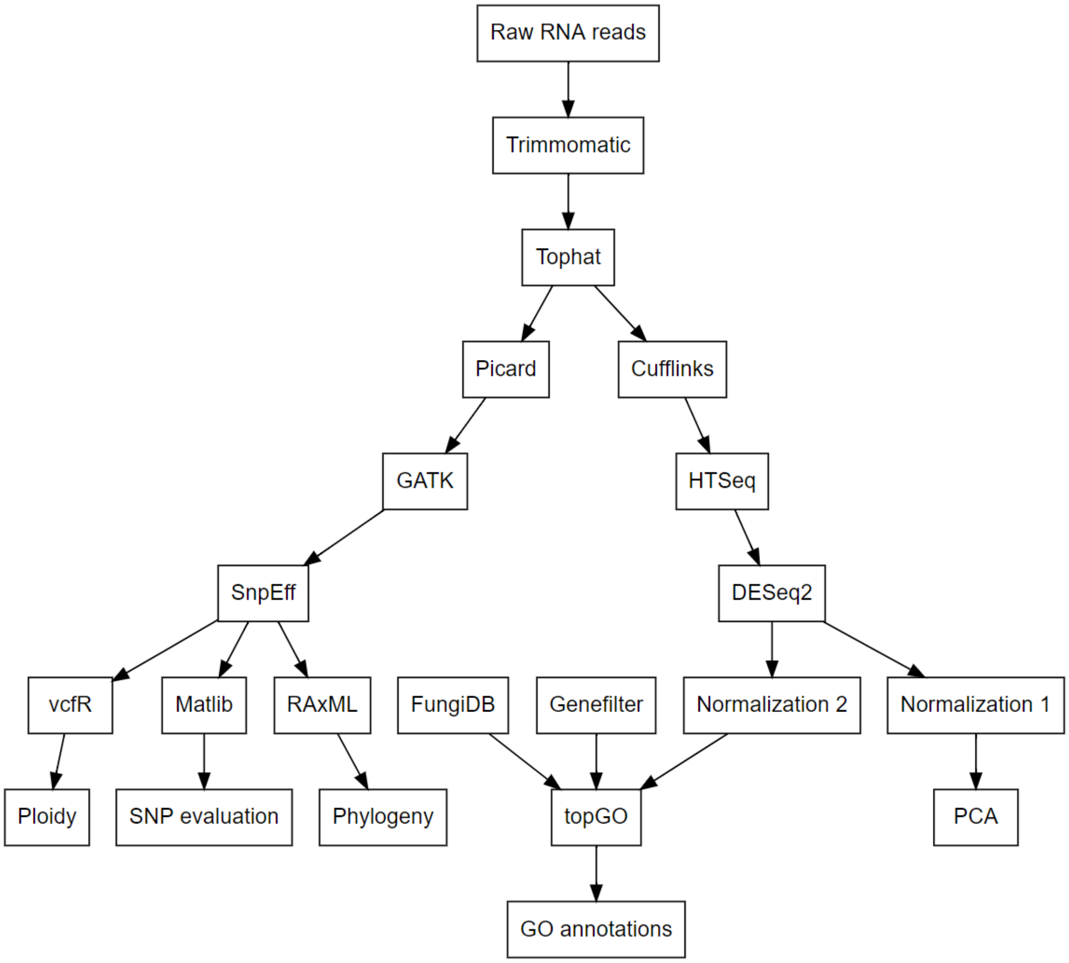

Supplementary figure 1. Illustration of the bioinformatic analysis. Trimmomatic was applied to trim for low quality reads and adaptor sequences on the 48 RNAseq samples (raw reads). Followed by mapping to the *P. infestans* genome (Tophat) and detection of novel transcripts (Cufflinks). To assess the read mapping, HTSeq count was run. DEseq2 was used with two different normalizations for the differential expression analysis. Normalization 1 was performed using data from strain 88069 and a principal component analysis (PCA) was applied on the resulting dataset. The second normalization was achieved by using both parental strains in each mating pair as normalization strains. Gene Ontology (GO) enrichment analysis was performed with topGO using GO-terms from FungiDB. The background set required for the GO enrichment analysis was selected with Genefinder, a function of the R package Genefilter. Picard, in combination with GATK functions SplitNCigarReads and Indel Realignment, was used to mark duplicated reads in the single nucleotide polymorphism (SNPs) analysis. This was followed by GATK HaplotypeCaller and GATK VariantFiltration to discover variants and filtering, respectively. To annotate the variants SnpEff was used. RAxML was run to perform the phylogenetic analysis and VcfR for the ploidy analysis. Calculations for the SNP based evaluation of the RNA content was done using Matlib.

```
T30-4        1 TT---TCCCTTTTCTTCCAGTTGGCAACAGATCTCCAAGCTCCCAAGCCTCCTACCCCCT
Swe3         1 TT---TCCCTTTTCTTCCAGTTGGCAACAGATCTCCAAGCTCCCAAGCCTCCTACCCCCT
Swe2         1 TT---TCCCTTTTCTTCCAGTTGGCAACAGATCTCCAAGCTCCCAAGCCTCCTACCCCCT
Swe1         1 AA-TTTCCCTTTTCTTCCAGTTGGCAACAGATCTCCAAGCTCCCAAGCCTCCTACCCCCT
Swe4         1 AAGTTTCCCTTTTCTTCCAGTTGGCAACAGATCTCCAAGCTCCCAAGCCTCCTACCCCCT
F80029       1 TT---TCCCTTTTCTTCCAGTTGGCAACAGATCTCCAAGCTCCCAAGCCTCCTACCCCCT
IPO82001     1 TT---TCCCTTTTCTTCCAGTTGGCAACAGATCTCCAAGCTCCCAAGCCTCCTACCCCCT
3928A        1 TT---TCCCTTTTCTTCCAGTTGGCAACAGATCTCCAAGCTCCCAAGCCTCCTACCCCCT
Pink6        1 TT---TCCCTTTTCTTCCAGTTGGCAACAGATCTCCAAGCTCCCAAGCCTCCTACCCCCT

T30-4       58 CCTTTAATCAAGATGGCTGACGATGATGTTCAAGCTTTGGTCGTTGACAACGGCTCCGGT
Swe3        58 CCTTTAATCAAGATGGCTGACGATGATGTTCAAGCTTTGGTCGTTGACAACGGCTCCGGT
Swe2        58 CCTTTAATCAAGATGGCTGACGATGATGTTCAAGCTTTGGTCGTTGACAACGGCTCCGGT
Swe1        60 CCTTTAATCAAGATGGCTGACGATGATGTTCAAGCTTTGGTCGTTGACAACGGCTCCGGT
Swe4        61 CCTTTAATCAAGATGGCTGACGATGATGTTCAAGCTTTGGTCGTTGACAACGGCTCCGGT
F80029      58 CCTTTAATCAAGATGGCTGACGATGATGTTCAAGCTTTGGTCGTTGACAACGGCTCCGGT
IPO82001    58 CCTTTAATCAAGATGGCTGACGATGATGTTCAAGCTTTGGTCGTTGACAACGGCTCCGGT
3928A       58 CCTTTAATCAAGATGGCTGACGATGATGTTCAAGCTTTGGTCGTTGACAACGGCTCCGGT
Pink6       58 CCTTTAATCAAGATGGCTGACGATGATGTTCAAGCTTTGGTCGTTGACAACGGCTCCGGT

T30-4      118 ATGTGCAAGGCCGGTTTCGCCGGTGACGACGCCCCGCGTGCCGTGTTCCCCTCGATTGTG
Swe3       118 ATGTGCAAGGCCGGTTTCGCCGGTGACGACGCCCCGCGTGCCGTGTTCCCCTCGATTGTG
Swe2       118 ATGTGCAAGGCCGGTTTCGCCGGTGACGACGCCCCGCGTGCCGTGTTCCCCTCGATTGTG
Swe1       120 ATGTGCAAGGCCGGTTTCGCCGGTGACGACGCCCCGCGTGCCGTGTTCCCCTCGATTGTG
Swe4       121 ATGTGCAAGGCCGGTTTCGCCGGTGACGACGCCCCGCGTGCCGTGTTCCCCTCGATTGTG
F80029     118 ATGTGCAAGGCCGGTTTCGCCGGTGACGACGCCCCGCGTGCCGTGTTCCCCTCGATTGTG
IPO82001   118 ATGTGCAAGGCCGGTTTCGCCGGTGACGACGCCCCGCGTGCCGTGTTCCCCTCGATTGTG
3928A      118 ATGTGCAAGGCCGGTTTCGCCGGTGACGACGCCCCGCGTGCCGTGTTCCCCTCGATTGTG
Pink6      118 ATGTGCAAGGCCGGTTTCGCCGGTGACGACGCCCCGCGTGCCGTGTTCCCCTCGATTGTG

T30-4      178 GGTCGCCCCAAGCACTTGGGAATCATGGTGGGCATGGACCAGAAGGATGCCTACGTCGGT
Swe3       178 GGTCGCCCCAAGCACTTGGGAATCATGGTGGGCATGGACCAGAAGGATGCCTACGTCGGT
Swe2       178 GGTCGCCCCAAGCACTTGGGAATCATGGTGGGCATGGACCAGAAGGATGCCTACGTCGGT
Swe1       180 GGTCGCCCCAAGCACTTGGGAATCATGGTGGGCATGGACCAGAAGGATGCCTACGTCGGT
Swe4       181 GGTCGCCCCAAGCACTTGGGAATCATGGTGGGCATGGACCAGAAGGATGCCTACGTCGGT
F80029     178 GGTCGCCCCAAGCACTTGGGAATCATGGTGGGCATGGACCAGAAGGATGCCTACGTCGGT
IPO82001   178 GGTCGCCCCAAGCACTTGGGAATCATGGTGGGCATGGACCAGAAGGATGCCTACGTCGGT
3928A      178 GGTCGCCCCAAGCACTTGGGAATCATGGTGGGCATGGACCAGAAGGATGCCTACGTCGGT
Pink6      178 GGTCGCCCCAAGCACTTGGGAATCATGGTGGGCATGGACCAGAAGGATGCCTACGTCGGT

T30-4      238 GACGAGGCCCAGTCCAAGCGTGGTGTGCTGACGCTTAAGTACCCTATTGAGCACGGTATT
Swe3       238 GACGAGGCCCAGTCCAAGCGTGGTGTGCTGACGCTTAAGTACCCTATTGAGCACGGTATT
Swe2       238 GACGAGGCCCAGTCCAAGCGTGGTGTGCTGACGCTTAAGTACCCTATTGAGCACGGTATT
Swe1       240 GACGAGGCCCAGTCCAAGCGTGGTGTGCTGACGCTTAAGTACCCTATTGAGCACGGTATT
Swe4       241 GACGAGGCCCAGTCCAAGCGTGGTGTGCTGACGCTTAAGTACCCTATTGAGCACGGTATT
F80029     238 GACGAGGCCCAGTCCAAGCGTGGTGTGCTGACGCTTAAGTACCCTATTGAGCACGGTATT
IPO82001   238 GACGAGGCCCAGTCCAAGCGTGGTGTGCTGACGCTTAAGTACCCTATTGAGCACGGTATT
3928A      238 GACGAGGCCCAGTCCAAGCGTGGTGTGCTGACGCTTAAGTACCCTATTGAGCACGGTATT
Pink6      238 GACGAGGCCCAGTCCAAGCGTGGTGTGCTGACGCTTAAGTACCCTATTGAGCACGGTATT

T30-4      298 GTGACCAACTGGGACGACATGGAGAAGATCTGGCACCACACCTTCTACAACGAGCTGCGT
Swe3       298 GTGACCAACTGGGACGACATGGAGAAGATCTGGCACCACACCTTCTACAACGAGCTGCGT
Swe2       298 GTGACCAACTGGGACGACATGGAGAAGATCTGGCACCACACCTTCTACAACGAGCTGCGT
Swe1       300 GTGACCAACTGGGACGACATGGAGAAGATCTGGCACCACACCTTCTACAACGAGCTGCGT
Swe4       301 GTGACCAACTGGGACGACATGGAGAAGATCTGGCACCACACCTTCTACAACGAGCTGCGT
F80029     298 GTGACCAACTGGGACGACATGGAGAAGATCTGGCACCACACCTTCTACAACGAGCTGCGT
IPO82001   298 GTGACCAACTGGGACGACATGGAGAAGATCTGGCACCACACCTTCTACAACGAGCTGCGT
3928A      298 GTGACCAACTGGGACGACATGGAGAAGATCTGGCACCACACCTTCTACAACGAGCTGCGT
Pink6      298 GTGACCAACTGGGACGACATGGAGAAGATCTGGCACCACACCTTCTACAACGAGCTGCGT
```

```
T30-4     358 GTGGCCCCCGAGGAGCACCCGGTGCTGCTTACCGAGGCCCCTCTTAACCCGAAGGCCAAC
Swe3      358 GTGGCCCCCGAGGAGCACCCGGTGCTGCTTACCGAGGCCCCTCTTAACCCGAAGGCCAAC
Swe2      358 GTGGCCCCCGAGGAGCACCCGGTGCTGCTTACCGAGGCCCCTCTTAACCCGAAGGCCAAC
Swe1      360 GTGGCCCCCGAGGAGCACCCGGTGCTGCTTACCGAGGCCCCTCTTAACCCGAAGGCCAAC
Swe4      361 GTGGCCCCCGAGGAGCCCCCGGTGCTGCTTACCGAGGCCCCTCTTAACCCGAAGGCCAAC
F80029    358 GTGGCCCCCGAGGAGCACCCGGTGCTGCTTACCGAGGCCCCTCTTAACCCGAAGGCCAAC
IPO82001  358 GTGGCCCCCGAGGAGCACCCGGTGCTGCTTACCGAGGCCCCTCTTAACCCGAAGGCCAAC
3928A     358 GTGGCCCCCGAGGAGCACCCGGTGCTGCTTACCGAGGCCCCTCTTAACCCGAAGGCCAAC
Pink6     358 GTGGCCCCCGAGGAGCACCCGGTGCTGCTTACCGAGGCCCCTCTTAACCCGAAGGCCAAC

T30-4     418 CGTGAGCGCATGACGCAGATCATGTTCGAGACGTTCAACGTGCCCGCCATGTATGTGAAC
Swe3      418 CGTGAGCGCATGACGCAGATCATGTTCGAGACGTGCAACGTGCCCGCCATGTATGTGAAC
Swe2      418 CGTGAGCGCATGACGCAGATCATGTTCGAGACGTTCAACGTGCCCGCCATGTATGTGAAC
Swe1      420 CGTGAGCGCATGACGCAGATCATGTTCGAGACGTTCAACGTGCCCGCCATGTATGTGAAC
Swe4      421 CGTGAGCGCATGACGCAGATCATGTTCGAGACGTTCAACGTGCCCGCCATGTATGTGAAC
F80029    418 CGTGAGCGCATGACGCAGATCATGTTCGAGACGTGCAACGTGCCCGCCATGTATGTGAAC
IPO82001  418 CGTGAGCGCATGACGCAGATCATGTTCGAGACGTTCAACGTGCCCGCCATGTATGTGAAC
3928A     418 CGTGAGCGCATGACGCAGATCATGTTCGAGACGTTCAACGTGCCCGCCATGTATGTGAAC
Pink6     418 CGTGAGCGCATGACGCAGATCATGTTCGAGACGTTCAACGTGCCCGCCATGTATGTGAAC

T30-4     478 ATCCAGGCCGTGCTGTCCCTGTACGCCTCTGGCCGTACCACGGGCTGTGTGCTCGACTCT
Swe3      478 ATCCAGGCCGTGCTGTCCCTGTACGCCTCTGGCCGTACCACGGGCTGTGTGCTCGACTCT
Swe2      478 ATCCAGGCCGTGCTGTCCCTGTACGCCTCTGGCCGTACCACGGGCTGTGTGCTCGACTCT
Swe1      480 ATCCAGGCCGTGCTGTCCCTGTACGCCTCTGGCCGTACCACGGGCTGTGTGCTCGACTCT
Swe4      481 ATCCAGGCCGTGCTGTCCCTGTACGCCTCTGGCCGTACCAGGGGCTGTGTGCTCGACTCT
F80029    478 ATCCAGGCCGTGCTGTCCCTGTACGCCTCTGGCCGTACCACGGGCTGTGTGCTCGACTCT
IPO82001  478 ATCCAGGCCGTGCTGTCCCTGTACGCCTCTGGCCGTACCACGGGCTGTGTGCTCGACTCT
3928A     478 ATCCAGGCCGTGCTGTCCCTGTACGCCTCTGGCCGTACCACGGGCTGTGTGCTCGACTCT
Pink6     478 ATCCAGGCCGTGCTGTCCCTGTACGCCTCTGGCCGTACCACGGGCTGTGTGCTCGACTCT

T30-4     538 GGTGACGGTGTGTCCCACACTGTGCCCATCTACGAGGGTTACGCTCTTCCTCACGCTATC
Swe3      538 GGTGACGGTGTGTCCCACACTGTGCCCATCTACGAGGGTTACGCTCTTCCTCACGCTATC
Swe2      538 GGTGACGGTGTGTCCCACACTGTGCCCATCTACGAGGGTTACGCTCTTCCTCACGCTATC
Swe1      540 GGTGACGGTGTGTCCCACACTGTGCCCATCTACGAGGGTTACGCTCTTCCTCACGCTATC
Swe4      541 GGTGACGGTGTGTCCCACACTGTGCCCATCTACGAGGGTTACGCTCTTCCTCACGCTATC
F80029    538 GGTGACGGTGTGTCCCACACTGTGCCCATCTACGAGGGTTACGCTCTTCCTCACGCTATC
IPO82001  538 GGTGACGGTGTGTCCCACACTGTGCCCATCTACGAGGGTTACGCTCTTCCTCACGCTATC
3928A     538 GGTGACGGTGTGTCCCACACTGTGCCCATCTACGAGGGTTACGCTCTTCCTCACGCTATC
Pink6     538 GGTGACGGTGTGTCCCACACTGTGCCCATCTACGAGGGTTACGCTCTTCCTCACGCTATC

T30-4     598 GTGCGTCTGGACCTGGCTGGCCGCGACCTGACGGACTACATGATGAAGATCCTGACGGAG
Swe3      598 GTGCGTCTGGACCTGGCTGGCCGCGACCTGACGGACTACATGATGAAGATCCTGAGGGAG
Swe2      598 GTGCGTCTGGACCTGGCTGGCCGCGACCTGACGGACTACATGATGAAGATCCTGAGGGAG
Swe1      600 GTGCGTCTGGACCTGGCTGGCCGCGACCTGACGGACTACATGATGAAGATCCTGACGGAG
Swe4      601 GTGCGTCTGGACCTGGCTGGCCGCGACCTGACGGACTACATGATGAAGATCCGGACGGAG
F80029    598 GTGCGTCTGGACCTGGCTGGCCGCGACCTGACGGACTACATGATGAAGATCCTGAGGGAG
IPO82001  598 GTGCGTCTGGACCTGGCTGGCCGCGACCTGACGGACTACATGATGAAGATCCTGAGGGAG
3928A     598 GTGCGTCTGGACCTGGCTGGCCGCGACCTGACGGACTACATGATGAAGATCCTGAGGGAG
Pink6     598 GTGCGTCTGGACCTGGCTGGCCGCGACCTGACGGACTACATGATGAAGATCCTGAGGGAG

T30-4     658 CGTGGTTACTCGTTCACGACCACGGCCGAGCGCGAAATTGTGCGTGACATCAAGGAGAAG
Swe3      658 CGTGGTTACTCGTTCACGACCACGGCCGAGCGCGAAATTGTGCGTGACATCAAGGAGAAG
Swe2      658 CGTGGTTACTCGTTCACGACCACGGCCGAGCGCGAAATTGTGCGTGACATCAAGGAGAAG
Swe1      660 CGTGGTTACTCGTTCACGACCACGGCCGAGCGCGAAATTGTGCGTGACATCAAGGAGAAG
Swe4      661 CGTGGTTACTCGTTCACGACCACGGCCGAGCGCGAAATTGTGCGTGACATCAAGGAGAAG
F80029    658 CGTGGTTACTCGTTCACGACCACGGCCGAGCGCGAAATTGTGCGTGACATCAAGGAGAAG
IPO82001  658 CGTGGTTACTCGTTCACGACCACGGCCGAGCGCGAAATTGTGCGTGACATCAAGGAGAAG
3928A     658 CGTGGTTACTCGTTCACGACCACGGCCGAGCGCGAAATTGTGCGTGACATCAAGGAGAAG
Pink6     658 CGTGGTTACTCGTTCACGACCACGGCCGAGCGCGAAATTGTGCGTGACATCAAGGAGAAG
```

```
T30-4     718  CTGACGTACATCGCTCTGGACTTCGACCAGGAGATGAAGACTGCCGCCGAGTCGTCGGGT
Swe3      718  CTGACGTACATCGCTCTGGACTTCGACCAGGAGATGAAGACTGCCGCCGAGTCGTCGGGT
Swe2      718  CTGACGTACATCGCTCTGGACTTCGACCAGGAGATGAAGACTGCCGCCGAGTCGTCGGGT
Swe1      720  CTGACGTACATCGCTCTGGACTTCGACCAGGAGATGAAGACTGCCGCCGAGTCGTCGGGT
Swe4      721  CTGACGTACATCGCTCTGGACTTCGACCAGGAGATGAAGACTGCCGCCGAGTCGTCGGGT
F80029    718  CTGACGTACATCGCTCTGGACTTCGACCAGGAGATGAAGACTGCCGCCGAGTCGTCGGGT
IPO82001  718  CTGACGTACATCGCTCTGGACTTCGACCAGGAGATGAAGACTGCCGCCGAGTCGTCGGGT
3928A     718  CTGACGTACATCGCTCTGGACTTCGACCAGGAGATGAAGACTGCCGCCGAGTCGTCGGGT
Pink6     718  CTGACGTACATCGCTCTGGACTTCGACCAGGAGATGAAGACTGCCGCCGAGTCGTCGGGT

T30-4     778  CTGGAGAAGAGCTACGAGCTGCCCGATGGCAACGTGATTGTCATCGGTAACGAGCGTTTC
Swe3      778  CTGGAGAAGAGCTACGAGCTGCCCGATGGCAACGTGATTGTCATCGGTAACGAGCGTTTC
Swe2      778  CTGGAGAAGAGCTACGAGCTGCCCGATGGCAACGTGATTGTCATCGGTAACGAGCGTTTC
Swe1      780  CTGGAGAAGAGCTACGAGCTGCCCGATGGCAACGTGATTGTCATCGGTAACGAGCGTTTC
Swe4      781  CTGGAGAAGAGCTACGAGCTGCCCGATGGCAACGTGATTGTCATCGGTAACGAGCGTTTC
F80029    778  CTGGAGAAGAGCTACGAGCTGCCCGATGGCAACGTGATTGTCATCGGTAACGAGCGTTTC
IPO82001  778  CTGGAGAAGAGCTACGAGCTGCCCGATGGCAACGTGATTGTCATCGGTAACGAGCGTTTC
3928A     778  CTGGAGAAGAGCTACGAGCTGCCCGATGGCAACGTGATTGTCATCGGTAACGAGCGTTTC
Pink6     778  CTGGAGAAGAGCTACGAGCTGCCCGATGGCAACGTGATTGTCATCGGTAACGAGCGTTTC

T30-4     838  CGTACCCCTGAGGTGCTGTTCCAGCCGTCGCTCATCGGTAAGGAAGCTTCGGGTATCCAC
Swe3      838  CGTACCCCTGAGGTGCTGTTCCAGCCGTCGCTCATCGGTAAGGAAGCTTCGGGTATCCAC
Swe2      838  CGTACCCCTGAGGTGCTGTTCCAGCCGTCGCTCATCGGTAAGGAAGCTTCGGGTATCCAC
Swe1      840  CGTACCCCTGAGGTGCTGTTCCAGCCGTCGCTCATCGGTAAGGAAGCTTCGGGTATCCAC
Swe4      841  CGTACCCCTGAGGTGCTGTTCCAGCCGTCGCTCATCGGTAAGGAAGCTTCGGGTATCCAC
F80029    838  CGTACCCCTGAGGTGCTGTTCCAGCCGTCGCTCATCGGTAAGGAAGCTTCGGGTATCCAC
IPO82001  838  CGTACCCCTGAGGTGCTGTTCCAGCCGTCGCTCATCGGTAAGGAAGCTTCGGGTATCCAC
3928A     838  CGTACCCCTGAGGTGCTGTTCCAGCCGTCGCTCATCGGTAAGGAAGCTTCGGGTATCCAC
Pink6     838  CGTACCCCTGAGGTGCTGTTCCAGCCGTCGCTCATCGGTAAGGAAGCTTCGGGTATCCAC

T30-4     898  GACTGCACGTTCCAGACCATCATGAAGTGTGACGTTGATATCCGTAAGGACTTGTACTGC
Swe3      898  GACTGCACGTTCCAGACCATCATGAAGTGTGACGTTGATATCCGTAAGGACTTGTACTGC
Swe2      898  GACTGCACGTTCCAGACCATCATGAAGTGTGACGTTGATATCCGTAAGGACTTGTACTGC
Swe1      900  GACTGCACGTTCCAGACCATCATGAAGTGTGACGTTGATATCCGTAAGGACTTGTACTGC
Swe4      901  GACTGCACGTTCCAGACCATCATGAAGTGTGACGTTGATATCCGTAAGGACTTGTACTGC
F80029    898  GACTGCACGTTCCAGACCATCATGAAGTGTGACGTTGATATCCGTAAGGACTTGTACTGC
IPO82001  898  GACTGCACGTTCCAGACCATCATGAAGTGTGACGTTGATATCCGTAAGGACTTGTACTGC
3928A     898  GACTGCACGTTCCAGACCATCATGAAGTGTGACGTTGATATCCGTAAGGACTTGTACTGC
Pink6     898  GACTGCACGTTCCAGACCATCATGAAGTGTGACGTTGATATCCGTAAGGACTTGTACTGC

T30-4     958  AACCACCATGAAGATCAAGGTGGTGGCCCCGCCTGAGCGCAAGTACTCGGTCTGGATCGG
Swe3      958  AACCACCATGAAGATCAAGGTGGTGGCCCCGCCTGAGCGCAAGTACTCGGTCTGGATCGG
Swe2      958  AACCACCATGAAGATCAAGGTGGTGGCCCCGCCTGAGCGCAAGTACTCGGTCTGGATCGG
Swe1      960  AACCACCATGAAGATCAAGGTGGTGGCCCCGCCTGAGCGCAAGTACTCGGTCTGGATCGG
Swe4      961  AACCACCATGAAGATCAAGGTGGTGGCCCCGCCTGAGCGCAAGTACTCGGTCTGGATCGG
F80029    958  AACCACCATGAAGATCAAGGTGGTGGCCCCGCCTGAGCGCAAGTACTCGGTCTGGATCGG
IPO82001  958  AACCACCATGAAGATCAAGGTGGTGGCCCCGCCTGAGCGCAAGTACTCGGTCTGGATCGG
3928A     958  AACCACCATGAAGATCAAGGTGGTGGCCCCGCCTGAGCGCAAGTACTCGGTCTGGATCGG
Pink6     958  AACCACCATGAAGATCAAGGTGGTGGCCCCGCCTGAGCGCAAGTACTCGGTCTGGATCGG

T30-4     1018 TGGTTCCATCCTGTCGTCGCTGTATTGTGCTCTCGGGTGGTACCACCATGTACCCGGGCA
Swe3      1018 TGGTTCCATCCTGTCGTCGCTGTATTGTGCTCTCGGGTGGTACCACCATGTACCCGGGCA
Swe2      1018 TGGTTCCATCCTGTCGTCGCTGTATTGTGCTCTCGGGTGGTACCACCATGTACCCGGGCA
Swe1      1020 TGGTTCCATCCTGTCGTCGCTGTATTGTGCTCTCGGGTGGTACCACCATGTACCCGGGCA
Swe4      1021 TGGTTCCATCCTGTCGTCGCTGTATTGTGCTCTCGGGTGGTACCACCATGTACCCGGGCA
F80029    1018 TGGTTCCATCCTGTCGTCGCTGTATTGTGCTCTCGGGTGGTACCACCATGTACCCGGGCA
IPO82001  1018 TGGTTCCATCCTGTCGTCGCTGTATTGTGCTCTCGGGTGGTACCACCATGTACCCGGGCA
3928A     1018 TGGTTCCATCCTGTCGTCGCTGTATTGTGCTCTCGGGTGGTACCACCATGTACCCGGGCA
Pink6     1018 TGGTTCCATCCTGTCGTCGCTGTATTGTGCTCTCGGGTGGTACCACCATGTACCCGGGCA
```

```
T30-4    1078 TTGGCGAGCGTATGACCAAGGAGCTTACGGCTCTGGCCCCGTCCGACGTTCCAGCAGATG
Swe3     1078 TTGGCGAGCGTATGACCAAGGAGCTTACGGCTCTGGCCCCGTCCGACGTTCCAGCAGATG
Swe2     1078 TTGGCGAGCGTATGACCAAGGAGCTTACGGCTCTGGCCCCGTCCGACGTTCCAGCAGATG
Swe1     1080 TTGGCGAGCGTATGACCAAGGAGCTTACGGCTCTGGCCCCGTCCGACGTTCCAGCAGATG
Swe4     1081 TTGGCGAGCGTATGACCAAGGAGCTTACGGCTCTGGCCCCGTCCGACGTTCCAGCAGATG
F80029   1078 TTGGCGAGCGTATGACCAAGGAGCTTACGGCTCTGGCCCCGTCCGACGTTCCAGCAGATG
IPO82001 1078 TTGGCGAGCGTATGACCAAGGAGCTTACGGCTCTGGCCCCGTCCGACGTTCCAGCAGATG
3928A    1078 TTGGCGAGCGTATGACCAAGGAGCTTACGGCTCTGGCCCCGTCCGACGTTCCAGCAGATG
Pink6    1078 TTGGCGAGCGTATGACCAAGGAGCTTACGGCTCTGGCCCCGTCCGACGTTCCAGCAGATG

T30-4    1138 TGGATCTCGAAGGCCGAGTACGACGAGTCTGGACCCTCGATCGTGCACCGCAAGTGCTTC
Swe3     1138 TGGATCTCGAAGGCCGAGTACGACGAGTCTGGACCCTCGATCGTGCACCGCAAGTGCTTC
Swe2     1138 TGGATCTCGAAGGCCGAGTACGACGAGTCTGGACCCTCGATCGTGCACCGCAAGGGCTTC
Swe1     1140 TGGATCTCGAAGGCCGAGTACGACGAGTCTGGACCCTCGATCGTGCACCGCAAGTGCTTC
Swe4     1141 TGGATCTCGAAGGCCGAGTACGACGAGTCTGGACCCTCGATCGTGCACCGCAAGTGCTTC
F80029   1138 TGGATCTCGAAGGCCGAGTACGACGAGTCTGGACCCTCGATCGTGCACCGCAAGTGCTTC
IPO82001 1138 TGGATCTCGAAGGCCGAGTACGACGAGTCTGGACCCTCGATCGTGCACCGCAAGTGCTTC
3928A    1138 TGGATCTCGAAGGCCGAGTACGACGAGTCTGGACCCTCGATCGTGCACCGCAAGTGCTTC
Pink6    1138 TGGATCTCGAAGGCCGAGTACGACGAGTCTGGACCCTCGATCGTGCACCGCAAGTGCTTC

T30-4    1198 TAAGCTGCCTGTCCACGGGTCAACTGGACTTAGAATCTGGAGGAGAAATGCCGCTGCTCA
Swe3     1198 TAAGCTGCCTGTCCACGGGTCAACTGGACTTAGAATCTGGAGGAGAAATGCCGCTGCTCA
Swe2     1198 TAAGCTGCCTGTCCACGGGTCAACTGGACTTAGAATCTGGAGGAGAAATGCCGCTGCTCA
Swe1     1200 TAAGCTGCCTGTCCACGGGTCAACTGGACTTAGAATCTGGAGGAGAAATGCCGCTGCTCA
Swe4     1201 TAAGCTGCCTGTCCACGGGTCAACTGGACTTAGAATCTGGAGGAGAAATGCCGCTGCTCA
F80029   1198 TAAGCTGCCTGTCCACGGGTCAACTGGACTTAGAATCTGGAGGAGAAATGCCGCTGCTCA
IPO82001 1198 TAAGCTGCCTGTCCACGGGTCAACTGGACTTAGAATCTGGAGGAGAAATGCCGCTGCTCA
3928A    1198 TAAGCTGCCTGTCCACGGGTCAACTGGACTTAGAATCTGGAGGAGAAATGCCGCTGCTCA
Pink6    1198 TAAGCTGCCTGTCCACGGGTCAACTGGACTTAGAATCTGGAGGAGAAATGCCGCTGCTCA

T30-4    1258 CGCGTGTGGCGTCTTGAGGCTTAAGTGCTTCGATGCACGCGGGTAAGTGGTGGTGCCGTC
Swe3     1258 CGCGTGTGGCGTCTTGAGGCTTAAGTGCTTCGATGCACGCGGGTAAGTGGTGGTGCCGTC
Swe2     1258 CGCGTGTGGCGTCTTGAGGCTTAAGTGCTTCGATGCACGCGGGTAAGTGGTGGTGCCGTC
Swe1     1260 CGCGTGTGGCGTCTTGAGGCTTAAGTGCTTCGATGCACCCGGGTAAGTGGTGGTGCCGTC
Swe4     1261 CGCGTGTGGCGTCTTGAGGCTTAAGTGCTTCGATGCACGCGGGTAAGTGGTGGTGCCGTC
F80029   1258 CGCGTGTGGCGTCTTGAGGCTTAAGTGCTTCGATGCACGCGGGTAAGTGGTGGTGCCGTC
IPO82001 1258 CGCGTGTGGCGTCTTGAGGCTTAAGTGCTTCGATGCACGCGGGTAAGTGGTGGTGCCGTC
3928A    1258 CGCGTGTGGCGTCTTGAGGCTTAAGTGCTTCGATGCACGCGGGTAAGTGGTGGTGCCGTC
Pink6    1258 CGCGTGTGGCGTCTTGAGGCTTAAGTGCTTCGATGCACGCGGGTAAGTGGTGGTGCCGTC

T30-4    1318 CATTTACTTTTCAAACCTTTCTTTGCTTTAATTTCTAAAATACTAGGATTGATGTTGTGA
Swe3     1318 CATTTACTTTTCAAACCTTTCTTTGCTTTAATTTCTAAAATACTAGGATTGATGTTGTGA
Swe2     1318 CATTTACTTTTCAAACCTTTCTTTGCTTTAATTTCTAAAATACTAGGATTGATGTTGTGA
Swe1     1320 CATTTACTTTTCAAACCTTTCTTTGCTTTAATTTCTAAAATACTAGGATTGATGTTGTGA
Swe4     1321 CATTTACTTTTCAAACCTTTCTTTGCTTTAATTTCTAAAATACTAGGATTGATGTTGTGA
F80029   1318 CATTTACTTTTCAAACCTTTCTTTGCTTTAATTTCTAAAATACTAGGATTGATGTTGTGA
IPO82001 1318 CATTTACTTTTCAAACCTTTCTTTGCTTTAATTTCTAAAATACTAGGATTGATGTTGTGA
3928A    1318 CATTTACTTTTCAAACCTTTCTTTGCTTTAATTTCTAAAATACTAGGATTGATGTTGTGA
Pink6    1318 CATTTACTTTTCAAACCTTTCTTTGCTTTAATTTCTAAAATACTAGGATTGATGTTGTGA

T30-4    1378 TCTATCC
Swe3     1378 TCTATCC
Swe2     1378 TCTATCC
Swe1     1380 TCTATCC
Swe4     1381 TCTATCC
F80029   1378 TCTATCC
IPO82001 1378 TCTATCC
3928A    1378 TCTATCC
Pink6    1378 TCTATCC
```

Supplementary figure 2. SNP analysis on *actA* gene *(PITG_15117).*
Sequence similarities are depicted with black. Analysis has been conducted by Clustal Omega algorithm

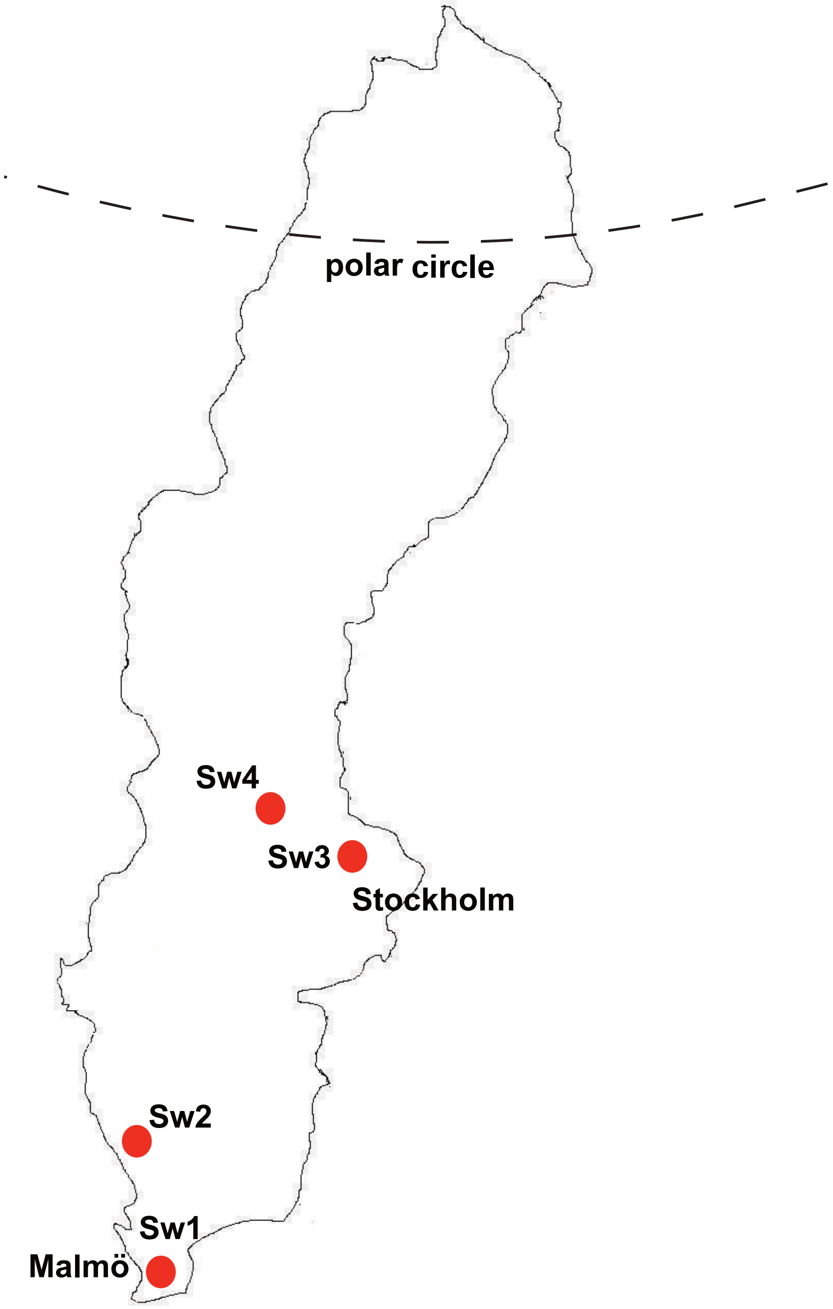

Supplementary figure 3. Map of Sweden showing the locations where Swedish isolates (Sw1-Sw4) have been collected.

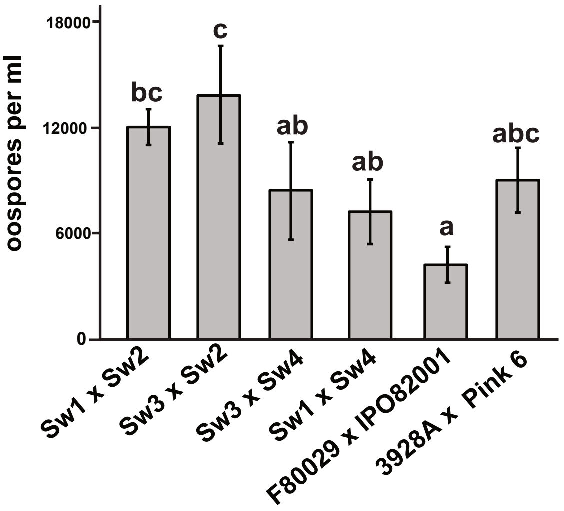

Supplementary figure 4. Oospore production during mating of Swedish, Dutch and British strains. Strains were grown on RPA media and materials from the mating zones were harvested 14 days post inoculation, suspended in 20 ml H20 and oospores were counted using hematocytometer. Error bars represent standard deviation (SD) based on at least three biological replicates. Different letters (a, b and c) indicate statistically significant differences according to Tukey's test ($p < 0.05$).

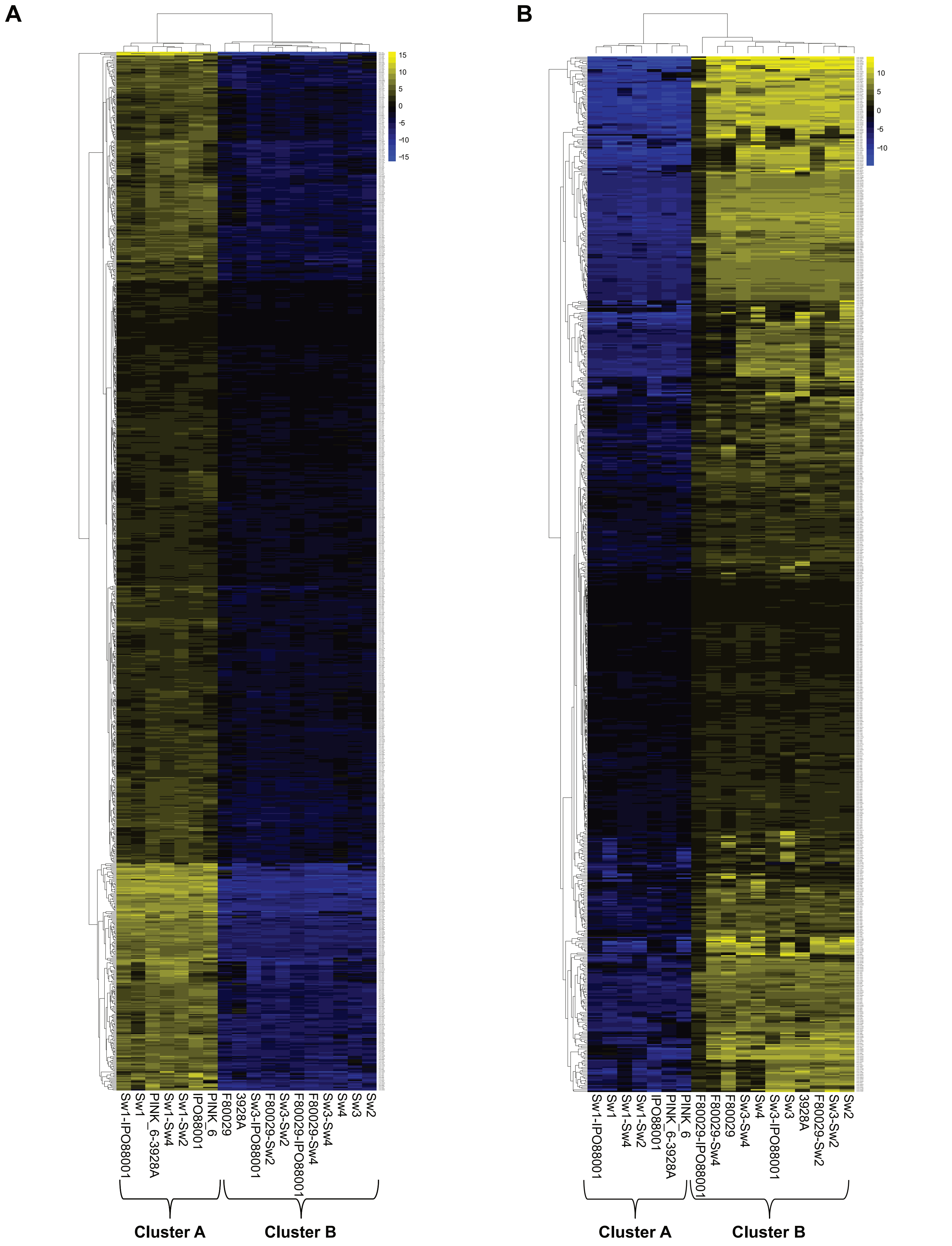

Supplementary figure 5. Transcription profiles of genes (**A**) up-regulated in PCA cluster A compared to cluster B (log2 fold change > 1) (**B**) up-regulated in PCA cluster B compared to cluster A (log2 fold change > 1). Data were normalized to the transcriptome of strain 88069 (Adjusted p-value <0.05). Yellow and blue colors represent up or down-regulated genes.

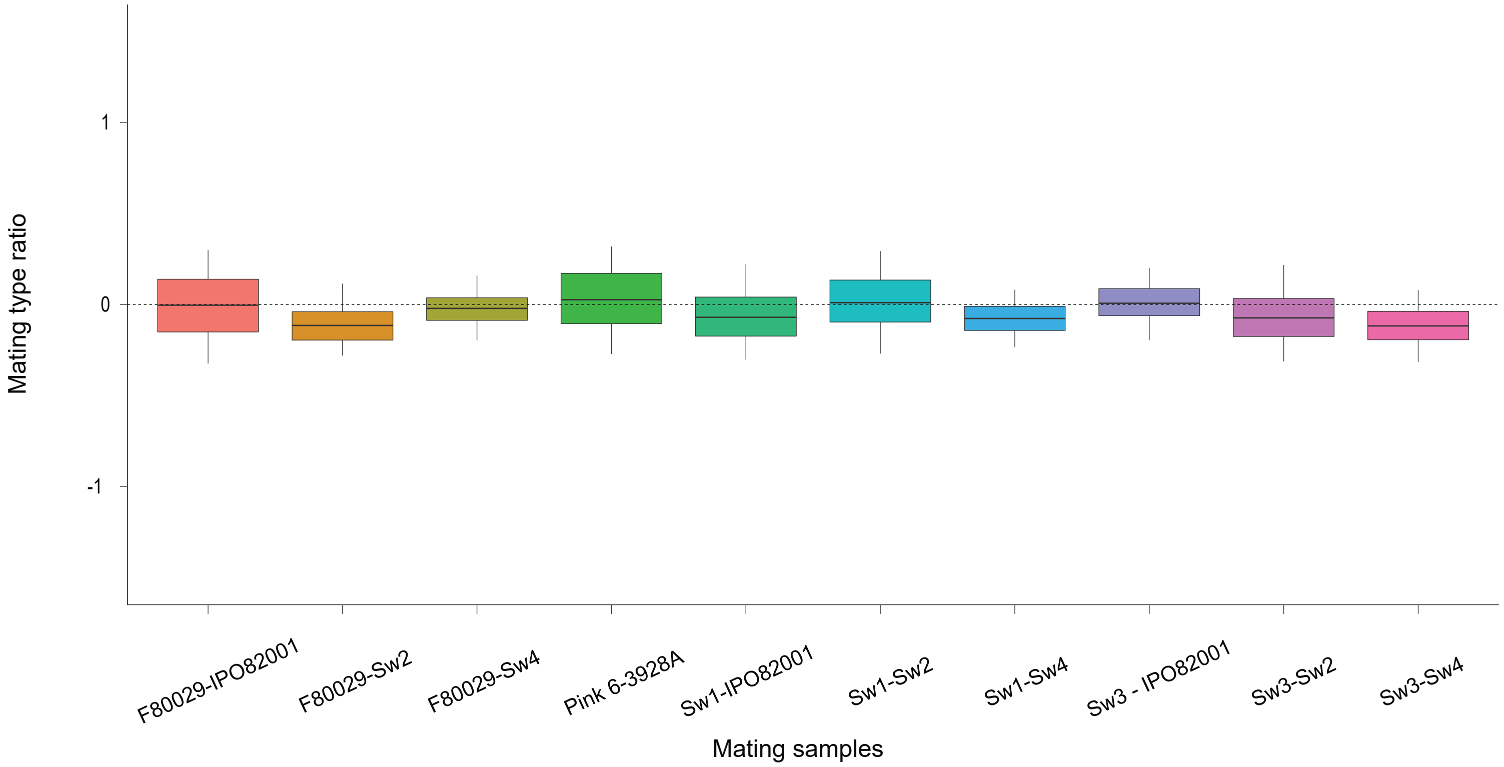

Supplementary figure 6. Estimation of RNA content based on SNP variation. Data is based on 14000 SNPs after filtering and outlier removal. The y-axis depicts the proportion of the mating types (A1/A2) in log_{10} scale. Boxes displays the lower quartile (Q1 = 25th percentile) and the upper quartile (Q3 = 75th percentile). The middle bar represents the median. The outer bars represent the lower limit (Q1 - 1.5 Interquartile range, IQR = Q3 - Q1) and upper limit (Q3 + 1.5 IQR). The analysis is made in R (version 3.5.2) using package Matlib (version 0.91).

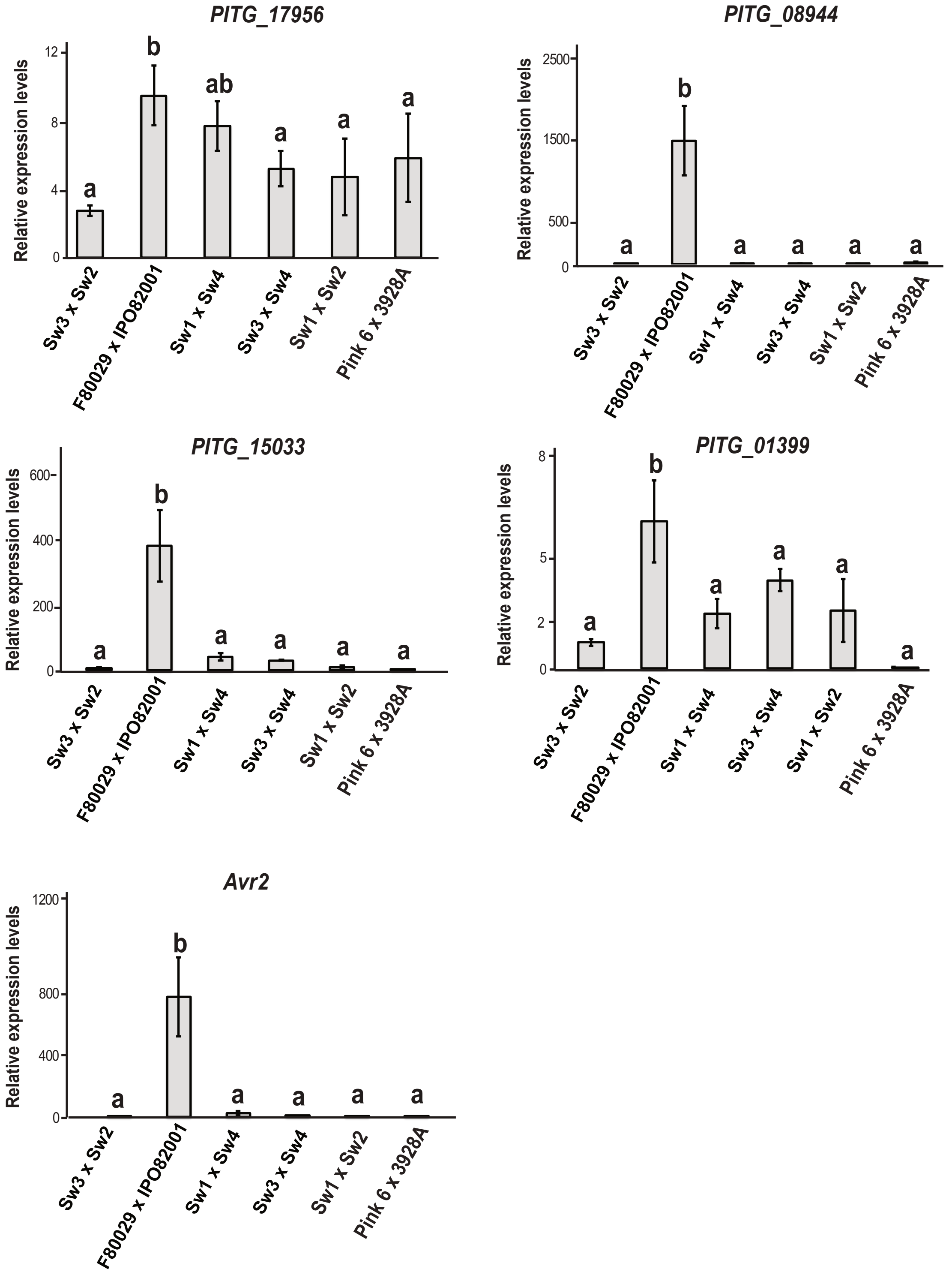

Supplementary figure 7 . Validation of RNA-seq analysis by qRT-PCR. Selected *P. infestans* genes up- regulated during mating between Swedish stains as compared to the Dutch and British ones, normalized to parental strains grown individually. Relative expression levels in relation to the actin gene (*act*) expression are calculated according to the 2^{-DDCt} - method. Error bars represent standard deviation (SD). Different letters (a,b) indicate statistically significant differences among the crossings according to Tukey’s test ($p < 0.05$).

```
PITG_18683-t26_     1 GC----------------------------------------------------------
PITG_04085-t26_     1 ATGCGCAGTATTCTCTACGCTGTTCTTGCCTTTGCGGTTTTAGCTAGGAGCAGCGCCGTC
PITG_04090-t26_     1 ATGCGCAGTTTTCTCTACGGTGTTCTTGCCTTTGCGGTTTTAGCTAGGAGCAGCGCCGTC
PITG_20300-t26_     1 ATGCGCAGTTTTCTCTACGGTGTTCTTGCCTTTGCGGTTTTAGCTAGGAGCAGCGCCGTC
PITG_20301-t26_     1 ATGCGCAGTATTCTCTACGCTGTTCTTGCCTTTGCGGTTTTAGCTAGGAGCAGCGCCGTC
PITG_20303-t26_     1 ATGCGCAGTATTCTCTACGCTGTTCTTGCCTTTGCGGTTTTAGCTAGGAGCAGCGCCGTC
PITG_04086-t26_     1 ATGCGCAGTATTCTCTACGCTGTTCTTGCCTTTGCGGTTTTAGCTAGGAGCAGCGCCGTC

PITG_18683-t26_     3 --AGCATTCCCAATCCCCGACGAGTCTCGCCCCTTGTCGAAGACATCTCCTGACACTGTG
PITG_04085-t26_    61 GCAGCATTCCCAATCCCCGACGAGTCTCGCCCCTTGTCGAAGACATCTCCTGACACTGTG
PITG_04090-t26_    61 GCAGCATTCCCAATCCCCGACGAGTCTCGCCCCTTGTCGAAGACATCTCCTGACACTGTG
PITG_20300-t26_    61 GCAGCATTCCCAATCCCCGACGAGTCTCGCCCCTTGTCGAAGACATCTCCTGACACTGTG
PITG_20301-t26_    61 GCAGCATTCACAATCCCCGACGAGTCTCGCCCCTTGTCGAAGACATCTCCTGACACTGGG
PITG_20303-t26_    61 GCAGCATTCCCAATCCCCGACGAGTCTCGCCCCTTGTCGAAGACATCTCCTGACACTGGG
PITG_04086-t26_    61 GCAGCATTCCCAATCCCCGACGAGTCTCGCCCCTTGTCGAAGACATCTCCTGACACTGTG

PITG_18683-t26_    61 GCCCCAAGATCGCTTCGGGTCGAGGCCCAAGAAGTTATTCAGAGCGGCCGGGGAGACGGA
PITG_04085-t26_   121 GCCACAAGATCGCTTCGGGTCGAGGCCCAAGAAGTTATTCAGAGCGGCCGGGGAGACGGA
PITG_04090-t26_   121 GCCCCAAGATCGCTTCGGATCGAGGCCCAAGAAGTTATTCAGAGCGGCCGGGGAGACGGA
PITG_20300-t26_   121 GCCCCAAGATCGCTTCGGATCGAGGCCCAAGAAGTTATTCAGAGCGGCCGGGGAGACGGA
PITG_20301-t26_   121 GCCACAAGATCGCTTCGGGTCGAGGCCCAAGAAGTTATTCAGAGCGGCCGGGGAGACGGA
PITG_20303-t26_   121 GCCACAAGATCGCTTCGGGTCGAGGCCCAAGAAGTTATTCAGAGCGGCCGGGGAGACGGA
PITG_04086-t26_   121 GCCACAAGATCGCTTCGGGTCGAGGCCCAAGAAGTTATTCAGAGCGGCCGGGGAGACGGA

PITG_18683-t26_   121 TATGGTGGGTTCTGGAAAAACATAATCCCGAGTACTAACAAGATCATCAAGAAGCCGGAT
PITG_04085-t26_   181 TATGGTGGGTTCTGGAAAAACATAATCCCGAGTACTAACAAGATCATCAAGAAGCCGGAT
PITG_04090-t26_   181 TATGGTGGGTTCTGGAAAAACGTAGCCCAGAGTACTAACAAGATCGTCAAGAGGCCGGAT
PITG_20300-t26_   181 TATGGTGGGTTCTGGAAAAACGTAGCCCAGAGTACTAACAAGATCGTCAAGAGGCCGGAT
PITG_20301-t26_   181 TATGGTGGGTTCTGGAAAAACGTTTTTCCGAGTACTAACAAGATCATCAAGAAGCCGGAT
PITG_20303-t26_   181 TATGGTGGGTTCTGGAAAAACGTTTTTCCGAGTACTAACAAGATCATCAAGAAGCCGGAT
PITG_04086-t26_   181 TATGGTGGGTTCTGGAAAAACATAATCCCGAGTACTAACAAGATCATCAAGAAGCCGGAT

PITG_18683-t26_   181 ATCAAGATAGGCAAACTTATCGAGGCGGCCAAGAAGGCAAAAAAAAAAATGACGAAGTCC
PITG_04085-t26_   241 ATCAAGATAAGCAAACTTATCGAGGCGGCCAAGAAGGCAAAAAAAAAAATGACGAAGTCC
PITG_04090-t26_   241 ATCAAGATAAGCAAACTTATCGCGGCGGCCAAGAAGGCAAAAGCAAAAATGACGAAGTCC
PITG_20300-t26_   241 ATCAAGATAGGCAAACTTATCGAGGCGGCCAAGAAGGCAAAAGCAAAAATGACGAAGTCC
PITG_20301-t26_   241 ATCAAGATAAGCAAACTTATCGCGGCGGCCAAGAAGGCAAAAGCAAAAATGACGAAGTCC
PITG_20303-t26_   241 ATCAAGATAAGCAAACTTATCGCGGCGGCCAAGAAGGCAAAAGCAAAAATGACGAAGTCC
PITG_04086-t26_   241 ATCAAGATAAGCAAACTTATCGAGGCGGCCAAGAAGGCAAAAAAAAAAATGACGAAGTCC

PITG_18683-t26_   241 TGA
PITG_04085-t26_   301 TGA
PITG_04090-t26_   301 TGA
PITG_20300-t26_   301 TGA
PITG_20301-t26_   301 TGA
PITG_20303-t26_   301 TGA
PITG_04086-t26_   301 TGA
```

Supplementary figure 8. Alignment of *Avrblb2* paralogs. Sequence similarities are depicted with black. Analysis has been conducted with the Clustal Omega algorithm.

A

SNPs analysis of Avrblb2 effector protein in *P. infestans* strains

Isolate name	Origin	Variant
Sw3	Sweden	Insertion
Sw2	Sweden	-
Sw1	Sweden	-
Sw4	Sweden	Deletion
F80029	Netherlands	Insertion
IPO82001	Netherlands	Deletion
Pink 6	UK	Deletion
3928A	UK	Insertion

B

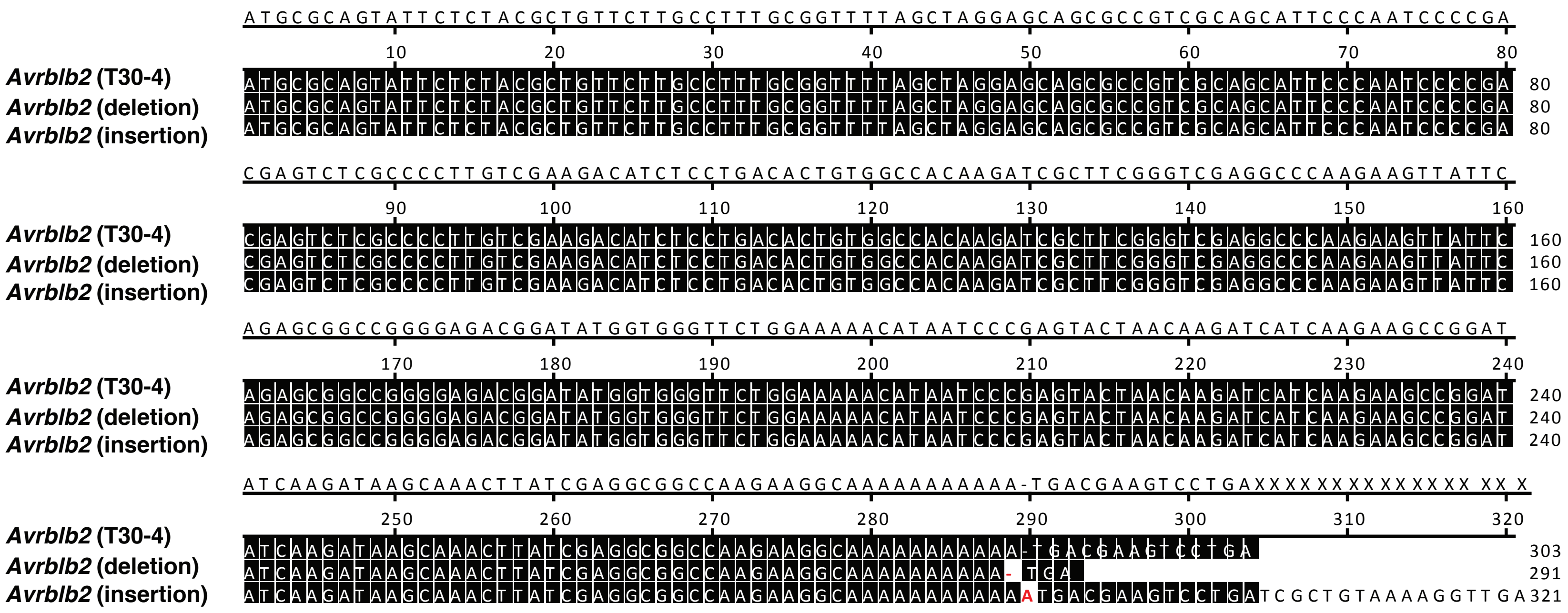

C

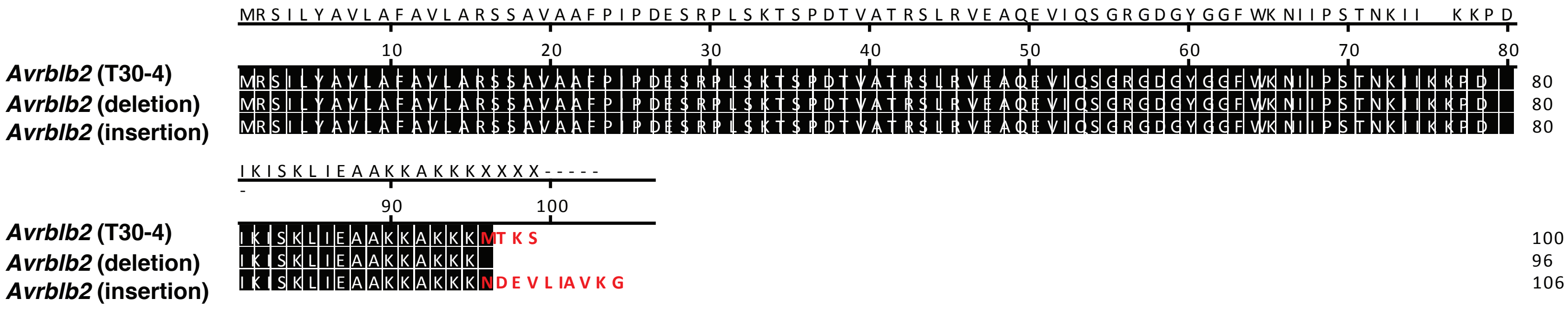

Supplementary figure 9. SNPs analysis of Avrblb2 effectors in *P. infestans* strains used in the current study. **A** table showing the insertions and deletions of nucleotides, **B** nucleotide alignment among different variants, **C** amino acid alignment among different variants

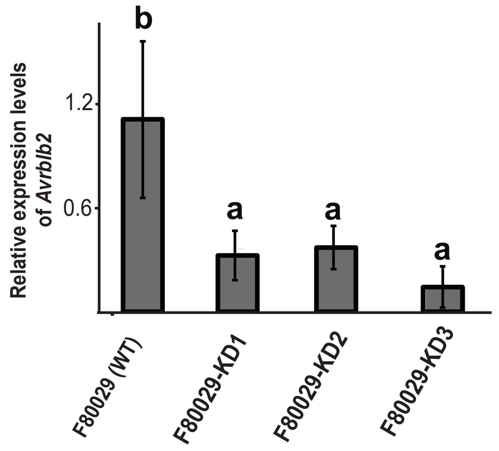

Supplementary figure 10. Validation of *P. infestans* Avrblb2 family knock-down (KD) transformants. Relative expression levels in relation to actin gene (act) expression were calculated according to the 2-DDCt method. Error bars represent SD based on at least three biological replicates. Different letters (a,b) indicate statistically significant differences between the F80029 strain and silenced lines according to Tukey's test ($p < 0.05$).

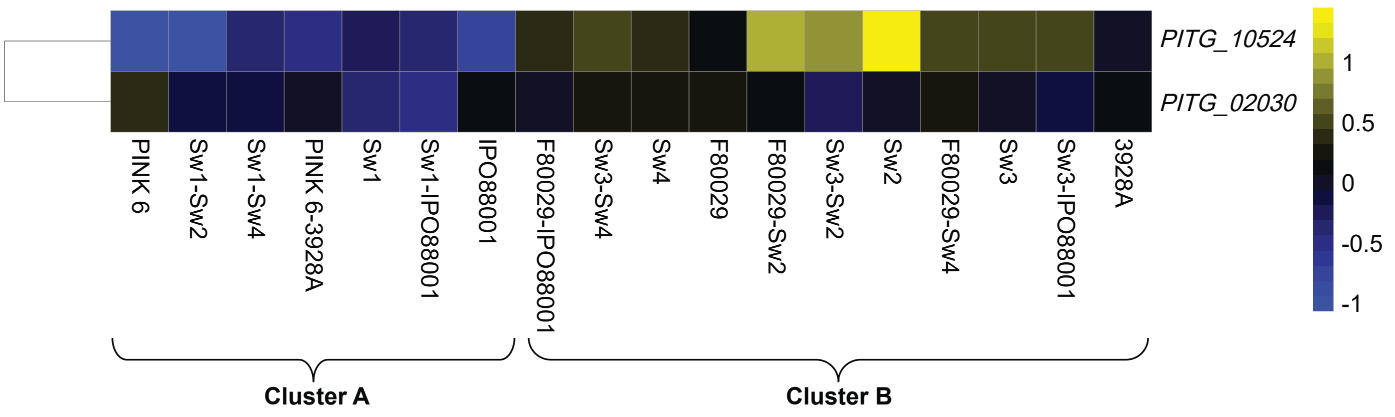

Supplementary figure 11. Transcription profiles of *PITG_02030* and *PITG_10524*. *P. infestans* genotypes are sorted according to the clustering of Avrblb2 (Fig. 4a). Data were normalized to the transcriptome of strain 88069 (PITG_10524 with adjusted p-value < 0.05). Yellow and blue colors represent up or down-regulated genes compared to strain 88069.
